# Candesartan, an angiotensin-II receptor blocker, ameliorates insulin resistance and hepatosteatosis by reducing intracellular calcium overload and lipid accumulation

**DOI:** 10.1038/s12276-023-00982-6

**Published:** 2023-05-01

**Authors:** Jin Wook Lee, Hyun-Oh Gu, Yunshin Jung, YunJae Jung, Seung-Yong Seo, Jeong-Hee Hong, In-Sun Hong, Dae Ho Lee, Ok-Hee Kim, Byung-Chul Oh

**Affiliations:** 1 Department of Physiology, Lee Gil Ya Cancer and Diabetes Institute, Gachon College of Medicine, Incheon, 21999 Republic of Korea; 2 Department of Health Sciences and Technology (GAIHST), Gachon University, Incheon, 21999 Republic of Korea; 3 Department of Microbiology, Lee Gil Ya Cancer and Diabetes Institute, Gachon University College of Medicine, Incheon, 21999 Republic of Korea; 4 College of Pharmacy, Gachon University, Incheon, 21936 Republic of Korea; 5 Department of Molecular Medicine, Lee Gil Ya Cancer and Diabetes Institute, Gachon University College of Medicine, Incheon, 21999 Republic of Korea; 6 Department of Internal Medicine, Gachon University Gil Medical Center, Incheon, 21565 Republic of Korea

**Keywords:** Insulin signalling, Obesity, Calcium signalling

## Abstract

Insulin resistance is a major contributor to the pathogenesis of several human diseases, including type 2 diabetes, hypertension, and hyperlipidemia. Notably, insulin resistance and hypertension share common abnormalities, including increased oxidative stress, inflammation, and organelle dysfunction. Recently, we showed that excess intracellular Ca^2+^, a known pathogenic factor in hypertension, acts as a critical negative regulator of insulin signaling by forming Ca^2+^-phosphoinositides that prevent the membrane localization of AKT, a key serine/threonine kinase signaling molecule. Whether preventing intracellular Ca^2+^ overload improves insulin sensitivity, however, has not yet been investigated. Here, we show that the antihypertensive agent candesartan, compared with other angiotensin-II receptor blockers, has previously unrecognized beneficial effects on attenuating insulin resistance. We found that candesartan markedly reduced palmitic acid (PA)-induced intracellular Ca^2+^ overload and lipid accumulation by normalizing dysregulated store-operated channel (SOC)-mediated Ca^2+^ entry into cells, which alleviated PA-induced insulin resistance by promoting insulin-stimulated AKT membrane localization and increased the phosphorylation of AKT and its downstream substrates. As pharmacological approaches to attenuate intracellular Ca^2+^ overload in vivo, administering candesartan to obese mice successfully decreased insulin resistance, hepatic steatosis, dyslipidemia, and tissue inflammation by inhibiting dysregulated SOC-mediated Ca^2+^ entry and ectopic lipid accumulation. The resulting alterations in the phosphorylation of key signaling molecules consequently alleviate impaired insulin signaling by increasing the postprandial membrane localization and phosphorylation of AKT. Thus, our findings provide robust evidence for the pleiotropic contribution of intracellular Ca^2+^ overload in the pathogenesis of insulin resistance and suggest that there are viable approved drugs that can be repurposed for the treatment of insulin resistance and hypertension.

## Insulin resistance: blocking calcium overload offers treatment option

A drug usually used to treat high blood pressure (hypertension) shows great promise in preventing insulin resistance, where cells no longer respond to insulin thereby increasing the risk of type 2 diabetes, by reducing intracellular calcium overload in cells. High intracellular calcium levels associated with obesity have been strongly linked to insulin resistance. Byung-Chul Oh and Ok-Hee Kim at Gachon College of Medicine in Incheon, South Korea, and co-workers investigated the effects of several anti-hypertension drugs in the hope that they might reduce insulin resistance. The drug candesartan blocked calcium ions from entering cells, preventing lipid accumulation and promoting the localization of a key insulin signalling molecule on cell membranes. Obese mice treated with candesartan showed considerably lower insulin resistance and tissue inflammation, suggesting that this established drug could be repurposed.

## Introduction

Insulin resistance is a complex pathological condition characterized by reduced insulin-stimulated glucose uptake in adipocytes and skeletal muscle, impaired physiological suppression of hepatic gluconeogenesis and lipolysis, and impaired glycogen synthesis^1–4^. Importantly, the functional defects associated with insulin resistance lead to elevated plasma levels of glucose and lipids^2,5,6^. Insulin resistance is likewise associated with several pathological cues, including oxidative stress^7^, inflammation^8^, dysregulated intracellular Ca^2+^ homeostasis^9^, endoplasmic reticulum stress^10^, and mitochondrial dysfunction^11,12^. Notably, obesity-associated increases in intracellular Ca^2+^ levels have emerged as a key pathological event in insulin resistance and type 2 diabetes^9,13,14^. Recently, we reported that excess intracellular Ca^2+^ induced by obesity tightly binds with plasma membrane phosphoinositides (PIPs) to form Ca^2+^-PIPs, which blocks the membrane targeting of various pleckstrin homology (PH) domains and disrupts insulin signaling^15^. Thus, excess intracellular Ca^2+^ is a critical negative regulator of insulin signaling. Despite recent evidence, however, no effective therapeutic approach to target insulin resistance caused by obesity-associated intracellular Ca^2+^ overload is currently available.

Insulin resistance and obesity-related hypertension often co-occur and are components of metabolic syndrome and cardiovascular disease^16–18^. Specifically, intracellular Ca^2+^ overload is reportedly an early event in hypertension, arteriosclerosis, and cardiovascular diseases^19^. Among the available antihypertensive drugs, the Ca^2+^ channel blocker verapamil—a common antihypertensive drug targeting L-type Ca^2+^ channels—was shown to prevent obesity- or streptozotocin (STZ)-induced diabetes by promoting functional beta cell mass^20^. In addition, verapamil stimulated insulin production in human subjects with recent-onset type 1 diabetes by preserving beta cell function^21^ and restored obesity-associated autophagy defects by reducing intracellular Ca^2+^ levels^13^. Furthermore, clinical studies have revealed that high-dose verapamil promotes insulin production in type 1 diabetes patients by delaying the loss of beta-cell function and decreasing insulin requirements^22^. Prior clinical findings suggest that Ca^2+^ channel blockers may have beneficial effects on both obesity-associated diabetes and type 1 diabetes; however, high-dose verapamil therapy causes cardiac side effects that render it unsuitable for use in patients with diabetes or insulin resistance.

Angiotensin-II receptor blockers (ARBs) are highly effective antihypertensive drugs^23^ that elicit additional benefits, including a reduced risk of new-onset diabetes^24^, suppression of albuminuria and diabetic kidney-disease progression^25^, and decreased serum uric acid levels^26^. Moreover, epidemiological and experimental studies have found that ARBs have beneficial effects on glucose homeostasis, which could prevent hepatic steatosis, improve insulin sensitivity^27–30^, and prevent new-onset type 2 diabetes^31^. These findings suggest that some ARBs could alleviate insulin resistance and enhance glucose and lipid metabolism. Nonetheless, whether blocking angiotensin-II receptors attenuates insulin resistance remains controversial^32^. Moreover, it is not clear whether the reported effects are due to the classic angiotensin receptor blocker properties of ARBs or to some other currently unknown effects of ARBs^33^.

In the current study, we investigated the effects of several ARBs on palmitic acid (PA)-induced insulin resistance^34^ in HepG2 cells and found that the ARB candesartan specifically and consistently improved insulin sensitivity and normalized PA-induced intracellular Ca^2+^ homeostasis in HepG2 cells. Furthermore, candesartan markedly improved glucose intolerance and normalized intracellular Ca^2+^ homeostasis, consequently alleviating the insulin resistance metabolic profile in mice fed a high-fat diet (HFD). Our results provide a rationale for normalizing intracellular Ca^2+^ homeostasis to alleviate insulin resistance. Candesartan has been safely used in the clinic to treat patients with hypertension; thus, our results suggest that candesartan could be a safe and effective therapeutic approach to treat obesity-associated insulin resistance and metabolic disease.

## Materials and methods

### Animal care and use

Male C57BL/6 mice obtained from Orient Bio Inc. (Korea) were studied under protocols approved by the animal ethics committee of the Gachon University Lee Gil Ya Cancer and Diabetes Institute (LCDI-2021-0002). After 1 week of adaptation to a control AIN93G diet, 8-week-old male mice were placed on a 60% high-fat diet (HFD) for 8 weeks and maintained on a 12 h light and dark cycle with free access to food and water. After 8 weeks of HFD feeding, the mice were placed in three groups of four mice per cage and administered daily oral gavage of vehicle or 0.5 mg/kg or 1.0 mg/kg candesartan for 3 weeks while receiving the same HFD. During the observation period, the mice were monitored for body weight and food intake every 3 days. Food intake was measured for each cage and divided by the number of mice to obtain the total amount of food consumed per mouse per week.

### Cell lines and cell culture conditions

Human HepG2 cells obtained from the American Type Culture Collection (ATCC, Manassas, VA, USA) were authenticated by short tandem repeat sequencing and were determined to be mycoplasma free. HepG2 cells were grown in DMEM (Welgene, Korea) supplemented with 10% fetal bovine serum (FBS) and 1× penicillin‒streptomycin. CHO-IR/IRS1 cells were grown in F12 medium supplemented with 10% FBS and 1× penicillin‒streptomycin. At 70–80% confluency, transient transfection of HepG2 cells or CHO-IR/IRS1 cells was performed using Lipofectamine 2000 (Invitrogen) with 1 μg or 10 μg of total DNA. Cells were maintained at 37 °C under 5% CO_2_.

### Angiotensin receptor blockers (ARBs)

Azilsartan (SML-0432. Sigma–Aldrich), candesartan (9003239, Cayman), eprosartan (E2535, Sigma–Aldrich), fimasartan (SML3256, Sigma–Aldrich Cayman), telmisartan (11615, Cayman), and valsartan (SML0142, Sigma–Aldrich) were dissolved in DMSO and used in in vitro studies. For in vivo studies, we used candesartan cilexetil (SML0245, Sigma–Aldrich) dissolved in PBS.

### Immunoblotting and antibodies

Tissues and cells were lysed with modified RIPA buffer (50 mM Tris-HCl, pH 7.4, 1% Triton X-100, 150 mM NaCl, 1 mM EDTA; Cell Signaling Technology) with freshly added 1 mM Na3VO4, 10 mM NaF, and protease inhibitor cocktail (Complete-Mini, Roche). Equal amounts of protein were resolved on 4–12% gels and electrophoretically transferred to polyvinylidene difluoride (PVDF) membranes. The immunoblot was visualized with horseradish peroxidase-conjugated secondary antibodies and enhanced chemiluminescence (*ECL kit, GE Healthcare Life Sciences*) using a charge-coupled device camera (Amerccham ImageQuant 800). The antibodies used for immunoblotting are listed in Supplementary Table 1.

### Immunohistochemistry (IHC)

Tissues were fixed in 10% neutral buffered formalin and embedded in paraffin. For liver and adipose sections, we used a horseradish peroxidase-conjugated secondary antibody to F4/80 (anti-rat IgG) with a 3,3-diamino-benzidine (DAB) tetrahydrochloride substrate (DAKO) as described previously^35^. All sections were counterstained with hematoxylin (Thermo Scientific). After mounting, the stained slides were captured with a 3DHISTECH scanner (Budapest, Hungary). DAB was quantified using NIH ImageJ software (https://imagej.nih.gov/ij/, 1997–2017). Data are presented as fold changes.

### Measurement of the intracellular Ca^2+^ concentration and lipid accumulation

For the intracellular Ca^2+^ concentration and lipid accumulation, HepG2 cells were plated onto slide glass; treated with 0.5 mM PA for 16 h; incubated with 4 μM Fura-2-AM (Teflabs), 5 μM Calbryte 630 AM, or 2 μM BODIPY for 30 min in PBS at 37 °C in the dark; and then washed for 10 min with PBS. Then, the cells were counterstained with 4,6-diamidino-2-phenylindole (DAPI) to visualize the nuclei. After mounting, the stained slides were imaged with a Zeiss LSM 700 or Zeiss LSM 980 laser-scanning confocal microscope (Carl Zeiss) at the Core-facility for Cells using in vivo imaging and were analyzed with ZEN 2010 Software (Carl Zeiss). Intracellular Ca^2+^ was quantified from the microscopy images using NIH ImageJ software (https://imagej.nih.gov/ij/, 1997–2017). Data are presented as fold changes.

### Gene set enrichment analysis (GSEA)

GSEA was performed on expressed genes according to the software manual^36,37^. Gene sets with a nominal *p* value < 0.05 and an FDR < 0.25 were considered significant. The exact *p* value and FDR q value for the GSEA can be found in the Source Data. All expressed genes were log2 transformed and centered, and unsupervised hierarchical clustering was performed using the k-means clustering method in Cluster 3.0 software^38^. Java TreeView (v.4.0) was used to visualize the clustered heatmaps.

### Statistical analysis

Sample sizes for all experiments were based on preliminary results and previous experience conducting related experiments. Power calculations were not used to determine sample sizes. Animals for each group of experiments were randomly assigned. No animals were excluded from the statistical analysis. Unless otherwise noted, all data are presented as the mean ± standard deviation. Statistical comparisons of groups were performed by unpaired Student’s *t* test or one-way ANOVA with Tukey’s post hoc test for multiple comparisons or the *Holm-Šídák’s* post hoc test for multiple comparisons. All data were subjected to D’Agostino and Pearson (*n* > 8) or Shapiro–Wilk (*n* < 8) normality tests before analysis. Groups that failed the normality test (*p* < 0.05) were subjected to an outlier test (ROUT; *Q* = 1%), as recommended to determine whether the outlier was responsible for the failure of the normality test. If the exclusion of outliers led to passing of the normality test and altered the statistical results, the exclusion was made; if it did not change the statistical outcome, no data were excluded from the group in question. The Kruskal–Wallis test was used for nonparametric statistical analysis of data that did not have a normal distribution. We performed data analyses and prepared graphs using GraphPad Prism 9.4 (GraphPad Software Inc., San Diego, CA).

## Results

### Certain ARBs can alleviate insulin resistance in PA-treated HepG2 cells by attenuating lipid accumulation and intracellular Ca^2+^ overload

**Fig. 1:**
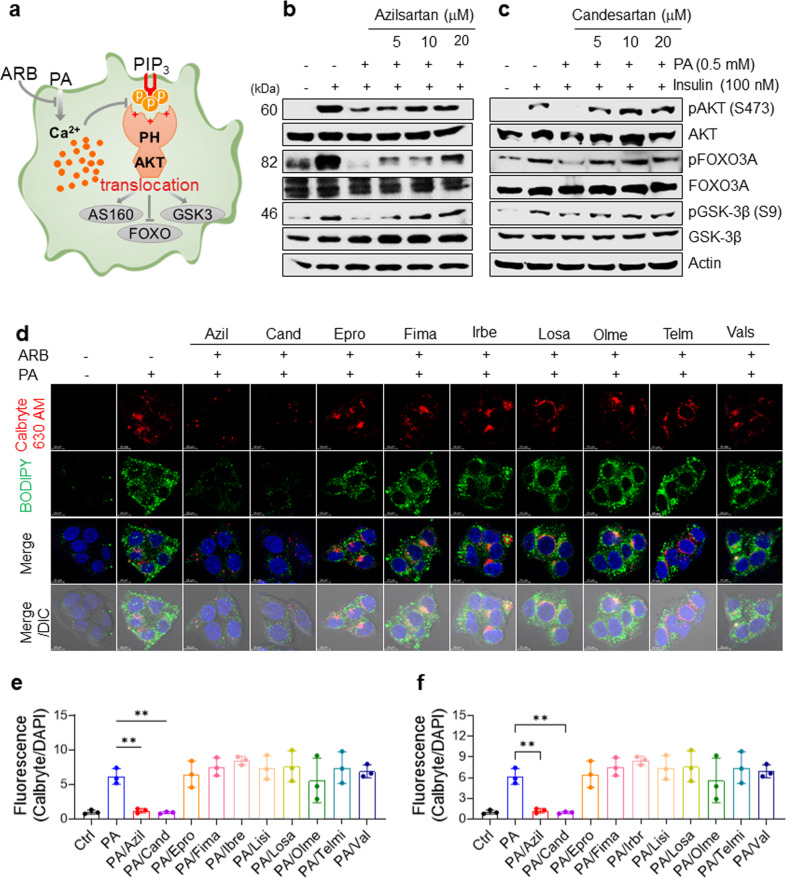
Certain ARBs can alleviate insulin resistance in PA-treated HepG2 cells by attenuating lipid accumulation and intracellular Ca^2+^ overload. **a** Schematic representation of the experimental hypothesis. **b**–**c** Immunoblots of lysates of human HepG2 cells treated with or without 0.5 mM PA and the indicated concentrations of ARBs for 16 h, followed by treatment with 100 nM insulin for 15 min. **d**–**f** Representative images of Calbryte 630 AM and BODIPY staining (**d**) and quantification of intracellular Ca^2+^ (**e**) and lipid accumulation (**f**) in HepG2 cells treated with vehicle, 0.5 mM PA, or 0.5 mM PA and the indicated ARBs. After the treatments, the cells were washed with PBS; stained for 30 min with 5 μM BODIPY, 5 μM Fluo-3 AM, or 5 μM Calbryte 630 AM and BODIPY in PBS; washed again with PBS; counterstained with 4,6-diamidino-2-phenylindole (DAPI) for nuclei; and observed using differential interference contrast (DIC) microscopy. Fluorescence intensities were quantified and normalized to the DAPI intensity using ImageJ (NIH, Bethesda, MD, USA).

Because excess intracellular Ca^2+^ inhibits insulin signaling by inhibiting PH-domain interactions with membrane PI(3,4)P_3_ or PI(3,4,5)P_3_, which abrogates membrane translocation of PH domains and leads to impaired insulin signaling^15^, we hypothesized that screening for biologically active compounds that restore impaired insulin signaling could identify potential drugs to ameliorate insulin resistance by attenuating intracellular Ca^2+^ overload (Fig. 1a). To assess whether and how ARBs restore impaired insulin signaling, we treated HepG2 cells for 16 h with 0.5 mM PA and three concentrations (5–20 μM) of nine FDA-approved ARBs that are commonly prescribed to treat hypertension in human patients. We then examined the insulin-stimulated phosphorylation of the crucial insulin signaling molecule Akt and the downstream signaling molecules GSK3β and FOXO3. Similar to our previous results^15^, PA treatment markedly attenuated the insulin-stimulated phosphorylation of Akt, GSK3β, and FOXO3 in HepG2 cells, confirming that PA treatment impairs insulin signaling in vitro. In addition, we found that the ARBs azilsartan and candesartan fully restored the insulin-stimulated phosphorylation of Akt, GSK3β, and FOXO3 in PA-treated HepG2 cells in a dose-dependent manner (Fig. 1b, c), whereas losartan, eprosartan, fimasartan, irbesartan, olmesartan, telmisartan, and valsartan did not have those effects (Supplementary Fig. 1a–g).

Next, we asked whether ARBs can modulate intracellular Ca^2+^ levels and lipid accumulation in PA-treated HepG2 cells. We tested the effects of the ARBs on the intracellular Ca^2+^ concentration and lipid accumulation in PA-treated HepG2 cells using confocal microscopy and the fluorescent dyes Calbryte 630 AM (for intracellular Ca^2+^) and BODIPY (for lipids). We found that azilsartan and candesartan significantly reduced the fluorescence intensity of intracellular Ca^2+^ (Fig. 1d, e) and lipid accumulation in PA-treated HepG2 cells (Fig. 1d, f), whereas eprosartan, fimasartan, irbesartan, losartan, olmesartan, and telmisartan did not affect the intracellular Ca^2+^ concentration or lipid accumulation in the PA-treated HepG2 cells (Fig. 1d–f). These results suggested that azilsartan and candesartan may restore impaired insulin signaling in PA-treated HepG2 cells by attenuating lipid accumulation and intracellular Ca^2+^ overload.

### Azilsartan and candesartan ameliorate insulin resistance by rescuing insulin-stimulated membrane recruitment of the AKT PH domain

**Fig. 2:**
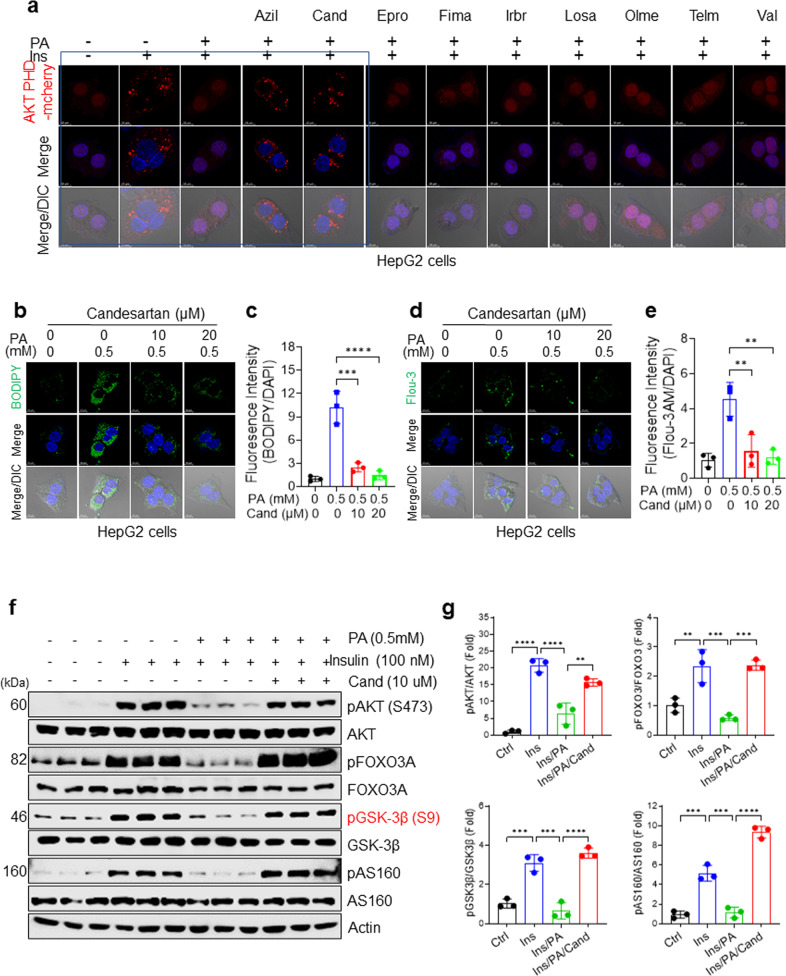
Azilsartan and candesartan ameliorate insulin resistance by rescuing insulin-stimulated membrane recruitment of the AKT PH domain. **a** Representative fluorescence images of Akt PH mCherry. HepG2 cells were transfected with the Akt PH mCherry fusion vector and treated for 16 h with 0.5 mM PA or 0.5 mM PA and the indicated ARBs. The cells were then serum-starved for 3 h, stimulated with 100 nM insulin for 15 min, counterstained with DAPI for nuclei, and observed using DIC microscopy. **b**, **c** Representative BODIPY images (**b**) and quantification (**c**) of intracellular Ca^2+^ in HepG2 cells treated with vehicle, 0.5 mM PA, or 0.5 mM PA and the indicated concentrations of candesartan for 16 h. **d**, **e** Representative Fluo-3 AM images (**d**) and quantification (**e**) of intracellular Ca^2+^ in HepG2 cells treated with vehicle, 0.5 mM PA, or 0.5 mM PA and the indicated concentrations of candesartan. All comparisons were made by one-way ANOVA with Tukey’s post hoc test. All data are presented as the mean ± SD (*n* = 3 per group). *, *p* < 0.05; **, *p* < 0.01; ***, *p* < 0.001, ****, *p* < 0.0001, compared with controls. **f** Immunoblot analysis of lysates of human HepG2 cells treated with 0.5 mM PA and 10 μM candesartan for 16 h followed by 100 nM insulin for 15 min. **g** The levels of phospho-proteins were quantified using ImageJ. All comparisons were made using one-way ANOVA with Tukey’s post hoc test. All data are presented as the mean ± standard deviation (SD) (*n* = 3 per group). *, *p* < 0.05; **, *p* < 0.01; ***, *p* < 0.001, ****, *p* < 0.0001, compared with controls.

The unique properties of azilsartan and candesartan to attenuate PA-induced intracellular Ca^2+^ overload raise the question of whether these ARBs modulate the subcellular localization of the AKT PH domain, which functions as a biosensor for PI(3,4,5)P_3_^39^. To address this question, we transiently expressed an Akt PH domain-mCherry (Akt PH mCherry) fusion vector in HepG2 cells and monitored its subcellular localization before and after treatment with ARBs in PA-treated HepG2 cells. AKT PH mCherry was promptly translocated from the cytosol to the plasma membrane after the cells were treated with 100 nM insulin for 15 min (Fig. 2a). Consistent with our previous findings^15^ that excess intracellular Ca^2+^ in hyperlipidemia acts as a negative regulator of Akt PH domain membrane localization, we confirmed that PA treatment inhibits insulin-stimulated membrane localization of the AKT PH domain (Fig. 2a). Treatment with azilsartan or candesartan rescued the PA-induced impairment of insulin-stimulated membrane recruitment of the AKT PH domain (Fig. 2a), endogenous AKT, and endogenous insulin receptor substrate 2 (IRS2, Supplementary Fig. 2a, b), suggesting a common mechanism rescuing impaired membrane targeting of PH domain-containing proteins such as AKT and IRS2. In contrast, treatments with the ARBs eprosartan, fimasartan, irbesartan, losartan, olmesartan, and telmisartan did not rescue the defective membrane recruitment of the AKT PH domain in PA-treated cells (Fig. 2a). We also confirmed that treatment with candesartan or azilsartan rescued insulin-stimulated AKT PH domain membrane localization in phorbol myristate acetate (PMA)-treated Chinese hamster ovary-insulin receptor (CHO-IR) cells (Supplementary Fig. 2c). These results demonstrate that azilsartan- or candesartan-mediated attenuation of intracellular Ca^2+^ overload can rescue the PA-induced impairment in insulin-stimulated membrane localization of the AKT PH domain.

Our results indicated that the ARBs azilsartan and candesartan had beneficial off-target effects on insulin signaling and intracellular Ca^2+^ homeostasis that are different from the known effects of ARBs against their intended biological target, AT1R^23^. Furthermore, although azilsartan and candesartan exhibited similar properties in restoring insulin signaling and intracellular Ca^2+^ homeostasis, we found that candesartan showed consistent insulin-sensitizing effects in a dose-dependent manner, which was not evident with azilsartan. Therefore, we focused on candesartan in further in-depth experiments. In addition, we validated the effects of candesartan on intracellular Ca^2+^ homeostasis and lipid accumulation using confocal microscopy with the fluorescent dyes BODIPY (for lipids) and Fluo-3AM (for intracellular Ca^2+^). We confirmed that candesartan significantly reduced PA-induced lipid accumulation and attenuated intracellular Ca^2+^ overload in HepG2 cells in a dose-dependent manner (Fig. 2b–e). Finally, to demonstrate enhanced insulin signaling, we conducted immunoblot analyses of AKT substrate of 160 (AS160)^40^ using an anti-phospho-AS160 antibody to test whether candesartan had beneficial effects on glucose uptake. We found that candesartan treatment increased the insulin-stimulated phosphorylation of AKT, FOXO3, GSK3β, and AS160 in PA-treated HepG2 cells compared with that in control cells (Fig. 2f, g). These results confirmed that candesartan fully restored impaired insulin signaling in PA-treated HepG2 cells.

### Candesartan normalizes dysregulated intracellular Ca^2+^ homeostasis by inhibiting store-operated Ca^2+^ entry in PA-treated HepG2 cells

**Fig. 3:**
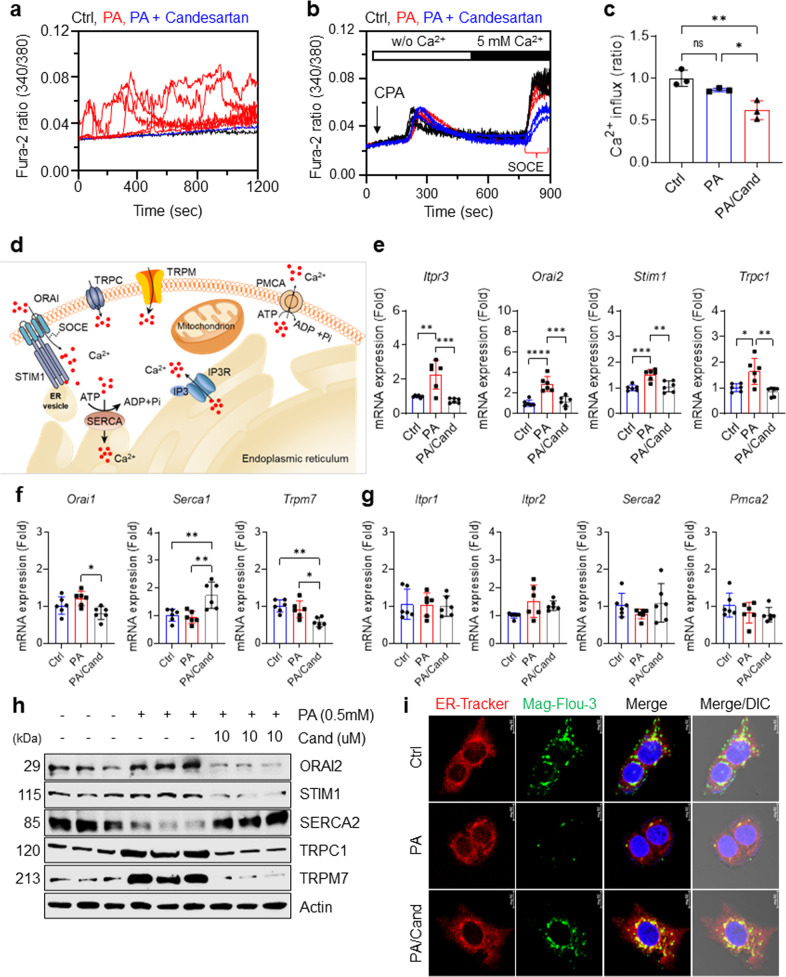
Candesartan normalizes dysregulated intracellular Ca^2+^ homeostasis by inhibiting store-operated Ca^2+^ entry (SOCE). **a** Mean traces for time-dependent changes in intracellular [Ca^2+^]i dynamics measured using the R340/380 fluorescence ratio in Fura-2 AM-loaded HepG2 cells treated for 24 h with vehicle, 0.5 mM PA, or 0.5 mM PA and 10 µM candesartan. **b** For measurement of SOCE, Fura-2 AM-loaded HepG2 cells were treated for 24 h with vehicle, 0.5 mM PA, or 0.5 mM PA and 10 µM candesartan; were stimulated with the SERCA inhibitor cyclopiazonic acid (CPA) in Ca^2+^-free solution to deplete ER Ca^2+^ stores; and were administered a 5 mM Ca^2+^ physiological salt solution. **c** The values of SOCE in the treated HepG2 cells were quantified by analysis of the fluorescence ratios at excitation wavelengths of 340 nm and 380 nm (340/380). **d** A schematic illustration of essential Ca^2+^ channels and transporters for intracellular Ca^2+^ homeostasis. **e** Relative mRNA expression of *Itpr3*, *Orai2*, *Stim1*, and *Trpc1* in HepG2 cells treated for 16 h with vehicle, 0.5 mM PA, or 0.5 mM PA and 10 μM candesartan. **f** Relative mRNA expression of *Orai1*, *Serca1*, and *Trpm7* in HepG2 cells treated for 16 h with vehicle, 0.5 mM PA, or 0.5 mM PA and 10 μM candesartan. **g** Relative mRNA expression of *Itpr1*, *Itpr2*, *Orai1*, *Serca1*, *Serca2*, *Pmca2*, and *Trpm7* in HepG2 cells treated for 16 h with vehicle, 0.5 mM PA, or 0.5 mM PA and 10 μM candesartan. **h** Immunoblot analysis of ORAI2, STIM1, SERCA2, TRPC1, and TRPM7 in the lysates of HepG2 cells treated for 16 h with vehicle, 0.5 mM PA, or 0.5 mM PA and 10 μM candesartan. **i** Representative Mag-Fluo-3 AM and ER tracker of HepG2 cells treated with vehicle, 0.5 mM PA, or 0.5 mM PA and 10 μM candesartan. HepG2 cells were incubated with the ER Ca^2+^ indicator Mag-Fluo-3 AM (5 μM) and ER Tracker (1 μM) for 2 h and visualized by confocal microscopy (scale bar 10 μm). Mag-Fluo-3 AM colocalized with the ER tracker in a punctate distribution, thereby reflecting ER Ca^2+^.

Next, we validated the effects of candesartan on intracellular Ca^2+^ homeostasis using a different fluorescent indicator, Fura-2 AM, to measure the intracellular Ca^2+^ concentration in HepG2 cells after 24 h of PA treatment. PA treatment led to sustained intracellular Ca^2+^ overload in HepG2 cells, which was significantly attenuated by candesartan treatment (Fig. 3a).

To gain insight into how candesartan suppresses intracellular Ca^2+^ overload, we determined the role of candesartan in Ca^2+^ entry into HepG2 cells via store-operated channels (SOCs)^41,42^ after depletion of cellular Ca^2+^ stores by cyclopiazonic acid (CPA), an inhibitor of the sarcoendoplasmic Ca^2+^-ATPase (SERCA)^43^ (Fig. 3b). We found that candesartan treatment effectively decreased SOC-mediated Ca^2+^ entry into PA-treated HepG2 cells (Fig. 3c), confirming that candesartan attenuates PA-induced intracellular Ca^2+^ overload by inhibiting dysregulated SOC-mediated Ca^2+^ entry (SOCE).

To assess how candesartan modulates intracellular Ca^2+^ levels in PA-treated HepG2 cells, we analyzed the expression levels of essential Ca^2+^ channels and transporters (Fig. 3d). PA treatment significantly upregulated the expression levels of inositol 1,4,5-trisphosphate receptor type 3 (*Itpr3*), *Orai2*, stromal interaction molecule 1 (*Stim1*), and transient receptor potential canonical 1 (*Trpc1*) compared with those in control cells (Fig. 3e). In line with our Ca^2+^ imaging results showing that candesartan inhibited SOC-mediated Ca^2+^ entry in PA-treated HepG2 cells, our qRT‒PCR studies showed that candesartan dramatically downregulated the expression of SOC-associated genes such as *Itpr3*, *Orai2*, *Stim1*, and *Trpc1* (Fig. 3e). Furthermore, candesartan treatment in PA-treated HepG2 cells upregulated the expression of Serca1 and downregulated the expression of *Orai1* and *Trpm7* (Fig. 3f), each of which encodes an essential Ca^2+^ channel or transporter involved in the regulation of intracellular Ca^2+^ concentration^44,45^. In contrast, PA treatment did not change the expression levels of calcium channels or transporters such as *Itpr1*, *Itpr2*, *Serca2*, and plasma membrane calcium-transporting ATPase 2 (*Pmca2*) compared with those in control cells or cells treated with both candesartan and PA (Fig. 3g). Immunoblot analysis further confirmed that candesartan normalized the expression of SOC proteins such as ORAI2, STIM1, TRPC1, and TRPM7 while markedly increasing the expression of SERCA2 in PA-treated HepG2 cells (Fig. 3h and Supplementary Fig. 3a). Moreover, candesartan may fully rescue ER Ca^2+^ stores by increasing ER membrane integrity and decreasing intracellular Ca^2+^ overload, as confirmed by confocal microscopy (Fig. 3i and Supplementary Fig. 3b, c). Overall, our findings suggest that candesartan treatment normalized dysregulated intracellular Ca^2+^ homeostasis in PA-treated HepG2 cells by regulating SOC-mediated Ca^2+^ entry into the cells.

### Candesartan ameliorates insulin resistance, hepatic steatosis, and dyslipidemia in HFD-fed mice

**Fig. 4:**
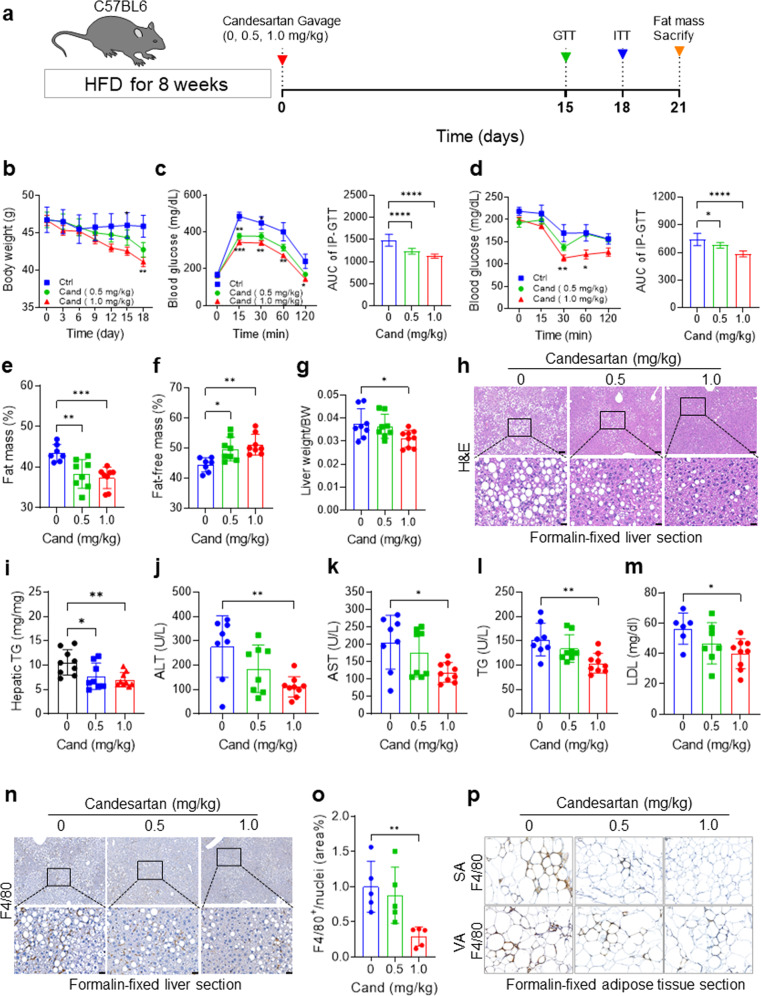
Candesartan improves insulin resistance, hepatic function, and steatosis in HFD-fed mice. **a** Schematic representation of the experimental design. After 1 week of adaptation to the chow diet, 8-week-old male C57BL/6 mice were fed a 60% HFD and were treated with vehicle or 0.5 mg/kg or 1.0 mg/kg candesartan for 21 days. **b** Mean body weight of the mice during the feeding period. **c** An intraperitoneal glucose tolerance test (IP-GTT) was conducted after 2 weeks of HFD feeding. The AUC of the IP-GTT results was calculated from the IP-GTT curve. **d** An intraperitoneal insulin tolerance test (IP-ITT) was conducted after 18 days of HFD feeding. The AUC of the IP-ITT results was calculated from the IP-ITT curve. **e**, **f** Whole-body fat mass (**e**) and fat-free lean mass (**f**) measured using a Minispec Live Mouse Analyzer LF50. (**g**) Liver mass relative to body mass. **h** Representative images of H&E staining of formalin-fixed liver sections. Scale bars = 200 µm and 50 µm. **i** The hepatic triglyceride contents were measured from liver lysates and normalized by dividing by the total protein content using a Beckman Coulter AU480 chemistry analyzer. **j**–**m** Serum levels of ALT (**j**), AST (**k**), T-CHO (**l**), and LDL (**m**) were measured with a Beckman Coulter AU480 chemistry analyzer. **n** Representative immunohistochemistry images of anti-F4/80 antibody in formalin-fixed liver sections. Scale bars = 200 µm and 50 µm. **o** The F4/80-positive area of liver sections was quantified using ImageJ. All comparisons were made by one-way ANOVA with Tukey’s post hoc test. All data are presented as the mean ± SD (*n* = 5–8 per group). *, *p* < 0.05; **, *p* < 0.01; ***, *p* < 0.001, ****, *p* < 0.0001, compared with controls. **p** Immunohistochemical staining of F4/80 showed that candesartan significantly reduced the expression of F4/80 in subcutaneous and visceral adipose tissues.

To evaluate the candesartan-mediated therapeutic potential of enhanced insulin signaling in vivo, we examined the effects of candesartan administration on insulin resistance and metabolic profiles in HFD-fed mice. After feeding 8-week-old male C57BL/6 mice a HFD for 8 weeks, we administered 0.5 mg/kg or 1.0 mg/kg candesartan per day for 21 days while continuing to provide the same HFD (Fig. 4a). The mice that received candesartan exhibited a gradual, dose-dependent decrease in body weight (Fig. 4b) without any change in food intake (Supplementary Fig. 4a) compared to that in the control group mice. In addition, the intraperitoneal glucose tolerance test (IP-GTT) and intraperitoneal insulin tolerance test (IP-ITT) showed significantly improved glucose tolerance and insulin sensitivity, respectively, with reduced areas under the curves (AUCs) in the mice that received candesartan compared with the control group mice (Fig. 4c, d). Furthermore, insulin resistance, as measured by the homeostatic model assessment for insulin resistance (HOMA-IR) index, was dramatically reduced in the mice that received candesartan compared to the control group mice (Supplementary Fig. 4b). These results demonstrated that candesartan improved glucose tolerance and insulin sensitivity in mice fed a HFD. Notably, mice that received 1.0 mg/kg candesartan daily exhibited a reduced ratio of whole-body fat mass to body weight (Fig. 4e) and an increased ratio of fat-free lean mass to body weight compared with control mice on a HFD, indicating that candesartan reduced fat mass without causing any changes in muscle mass (Fig. 4f).

In addition, the administration of candesartan decreased the ratio of liver weight to body weight (Fig. 4g) and improved biochemical and histological hepatic parameters, as confirmed by H&E staining of liver sections (Fig. 4h), quantification of hepatic triglyceride contents (Fig. 4i), and measurements of the liver function markers serum alanine transaminase (ALT) and aspartate transaminase (AST) (Fig. 4j, k). Furthermore, lipid profile analysis showed that candesartan markedly decreased the serum levels of triglycerides (Fig. 4l) and low-density lipoprotein cholesterol (LDL-C; Fig. 4m). qRT‒PCR analysis of hepatic gene expression demonstrated that candesartan reduced the expression of inflammatory genes such as *Ccl2*, *F4/80*, *Il-1β*, and *Tnf-α* (Supplementary Fig. 4c). Consistently, immunohistochemical staining of the macrophage marker F4/80 showed that candesartan treatment markedly reduced inflammatory macrophage infiltration and hepatic inflammation compared with the control mice fed a HFD (Fig. 4n,o). In adipose tissue, qRT‒PCR analysis showed that candesartan reduced the expression of F4/80 in subcutaneous and visceral adipose tissues (Supplementary Fig. 4d, e). Immunohistochemical staining of F4/80 further confirmed that the ratio of F4/80^+^ crown-like structures, a marker of metabolic inflammation, was reduced in subcutaneous and visceral adipose tissues after candesartan administration (Fig. 4p; Supplementary Fig. 4f, g). Moreover, we found that candesartan reduced the serum levels of creatinine and uric acid compared with the control mice fed a HFD (Supplementary Fig. 4h, i), suggesting that candesartan improved the renal function of mice fed a HFD.

### Candesartan alters the hepatic metabolism transcriptome

**Fig. 5:**
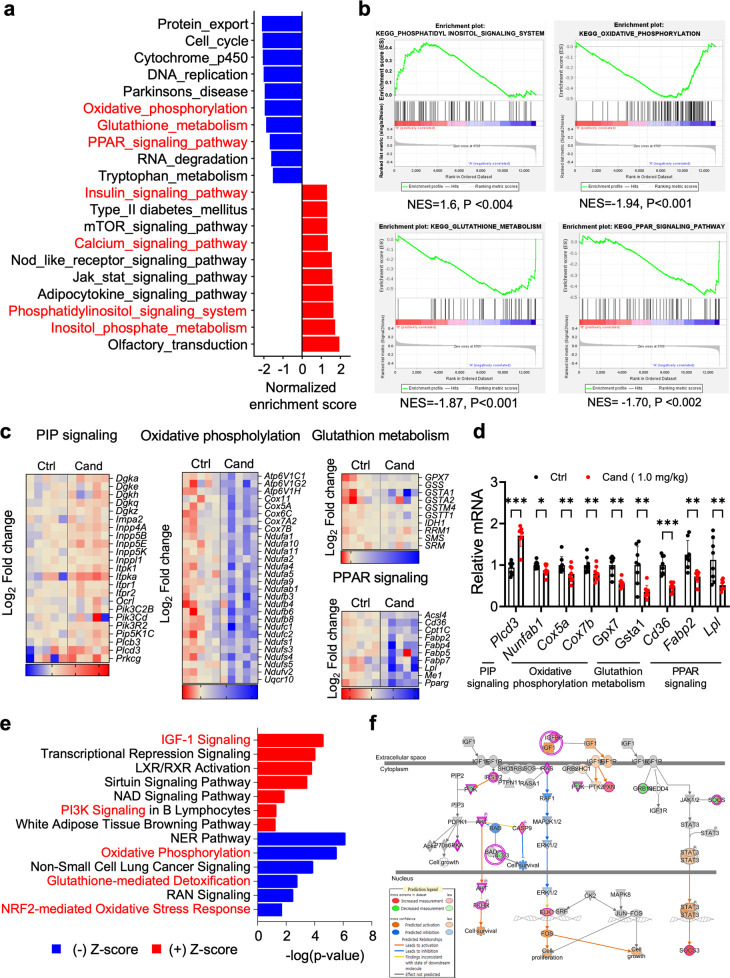
Candesartan alters the hepatic metabolism transcriptome. **a** Gene set enrichment analysis (GSEA) with KEGG gene sets revealed alterations in molecular pathways related to essential metabolic pathways in mice that received candesartan compared with control mice. **b** Enrichment plot showing the downregulation of the PPAR signaling pathway and upregulation of the PIP, JAK/STAT, and calcium signaling pathways. The GSEA permutation test yielded a *p* value < 0.05. **c** Functional clustering heatmap of differentially expressed genes. **d** qRT‒PCR of genes associated with the PIP signaling pathway, oxidative phosphorylation, and glutathione metabolism in control mice and mice that received candesartan. All comparisons were made by one-way ANOVA with Tukey’s post hoc test. All data are presented as the mean ± SD (*n* = 5–8 per group). *, *p* < 0.05; **, *p* < 0.01; ***, *p* < 0.001, ****, *p* < 0.0001, compared with controls. **e** IPA shows the downregulation of the oxidative phosphorylation, glutathione-mediated detoxification, and upregulation of the IGF1, PI3K, and NAD signaling pathways. **f** The signaling pathway of IGF1 shows that the pink circle represents upregulated genes.

To determine the molecular mechanisms underlying the enhanced metabolic profiles observed in mice that received candesartan, we performed RNA-seq on liver tissues from control and candesartan-treated mice. Gene expression analyses revealed 2,384 differentially expressed genes (*p* < 0.05), with 1017 genes increased and 1367 genes reduced in the mice that received candesartan compared with the control group mice (Supplementary Fig. 5a). Gene set enrichment analysis (GSEA) identified Kyoto Encyclopedia of Genes and Genomes (KEGG) and gene ontology (GO) terms that were enriched for upregulated or downregulated genes (Fig. 5a and Supplementary Fig. 5b). The KEGG terms for downregulated genes enriched in the mice that received candesartan compared with the control group mice included terms related to oxidative phosphorylation, glutathione metabolism, and the PPAR signaling pathway (Fig. 5a). Conversely, terms related to upregulated genes that were enriched in the mice that received candesartan compared with control mice included inositol phosphate metabolism response and PIP, JAK-STAT, and calcium signaling (Fig. 5a), indicating an increase in essential metabolic pathways. Interestingly, our GSEA revealed that the “oxidative phosphorylation signature” and “PPAR signaling signature” were markedly downregulated, whereas the “PIP signaling system signature” and “calcium signaling signature” were significantly upregulated in the mice administered candesartan compared with control mice (Fig. 5b and Supplementary Fig. 6a). Moreover, we confirmed that the heatmap plots of each pathway were differentially expressed in mice that received candesartan compared with control mice (Fig. 5c and Supplementary Fig. 6b). Using qRT‒PCR, we verified the dysregulated expression of select genes related to PIP signaling, oxidative phosphorylation, PPAR signaling, and glutathione metabolism (Fig. 5d). Consistently, ingenuity pathway analysis (IPA) revealed that IGF-1 signaling, LXR/RXR activation, PI3K signaling, protein kinase A, and calcium signaling were upregulated, whereas oxidative phosphorylation, glutathione-mediated detoxification, and NRF2-mediated oxidative stress response were downregulated (Fig. 5e). IPA analysis of transcriptome data indicated changes in several hepatotoxicity-related pathways, including hyperplasia and steatosis, after treating mice fed a HFD with 1 mg/kg candesartan (Supplementary Fig. 7a–c). Collectively, the results of our analyses revealed the distinct effects of candesartan treatment on gene expression and signaling pathways associated with insulin signaling (Fig. 5f) in mice fed a HFD.

### Candesartan modulates genes and proteins associated with SOCE in HFD-fed mice

**Fig. 6:**
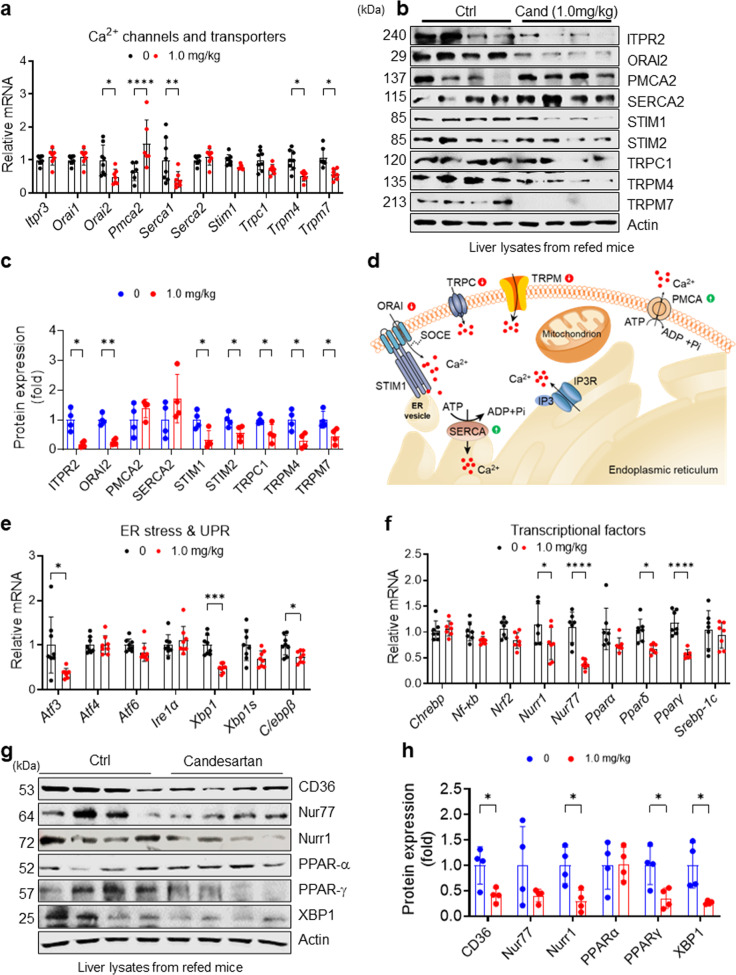
Candesartan modulates genes and proteins associated with intracellular Ca^2+^ homeostasis and metabolic activity in vivo. **a** Relative mRNA expression of Ca^2+^ channels and transporters in the livers of mice fed a 60% HFD and treated with vehicle or 1.0 mg/kg candesartan. **b** Immunoblot analysis of liver extracts from the mice after 16 h of overnight fasting and subsequent refeeding with HFD for 4 h. **c** Proteins in the immunoblots in (B) were quantified using ImageJ. **d** A schematic illustration of the effects of candesartan on the expression of essential Ca^2+^ channels and transporters for intracellular Ca^2+^ homeostasis. **e** Relative mRNA expression of genes associated with ER stress and the UPR in the livers of mice fed a 60% HFD and treated with vehicle or 1.0 mg/kg candesartan. **f** Relative mRNA expression of transcription factors associated with metabolism in the livers of mice fed a 60% HFD and treated with vehicle or 1.0 mg/kg candesartan. **g** Immunoblot analysis of liver extracts from mice after 16 h of overnight fasting and subsequent refeeding with HFD for 4 h. **h** Proteins in the immunoblot in (**g**) were quantified using ImageJ. All comparisons were conducted by two-way ANOVA with the Holm*-*Šídák’s post hoc test. All data are presented as the mean ± SD (*n* = 5–8 per group). *, *p* < 0.05; **, *p* < 0.01; ***, *p* < 0.001, ****, *p* < 0.0001, compared with controls.

To examine whether candesartan differentially regulates genes related to hepatic Ca^2+^ homeostasis in HFD-fed mice, we analyzed the hepatic expression levels of essential Ca^2+^ channel and transporter genes. Consistent with our in vitro findings, candesartan treatment in HFD-fed mice dramatically downregulated the expression of Ca^2+^ channel and transporter genes such as *Orai2*, *Serca1*, *Trpm4*, and *Trpm7* (Fig. 6a), the expression of which is known to increase intracellular Ca^2+^ concentrations^9,46^. Candesartan also reduced the expression of Stim1 and Trpc1 in liver tissues, but these changes were not statistically significant (Fig. 6a). In contrast with the downregulation of genes involved in increasing intracellular Ca^2+^ concentrations, candesartan upregulated the expression of *Pmca2*, the plasma membrane Ca^2+^ ATPase that transports intracellular Ca^2+^ ions to the extracellular compartment^47^ (Fig. 6a). Immunoblot analysis further confirmed that candesartan reduced the levels of ORAI2, STIM1, and TRPM7 compared with those in liver tissues from control mice (Fig. 6b, c), whereas it increased the levels of PMCA2 and SERCA2, although these increases were not statistically significant (Fig. 6b, c). Taken together, these results suggest that candesartan normalizes dysregulated intracellular Ca^2+^ homeostasis in HFD-fed mice by downregulating the expression of genes associated with SOCE, such as *Orai2*, *Stim1*, *Trpc1*, *Trpm4*, and *Trpm7*, or by upregulating the expression of *Pmca2* and *Serca2* (Fig. 6d).

### Candesartan modulates genes and proteins associated with ER stress and glucose and lipid metabolism in HFD-fed mice

Because disruption of Ca^2+^ homeostasis in obesity is an important cause of ER stress and impaired protein folding and carbohydrate and lipid metabolism^48^ and because candesartan normalized dysregulated intracellular Ca^2+^ homeostasis in the livers of HFD-fed mice, we evaluated the expression of genes associated with ER stress and glucose and lipid metabolism as well as their essential transcription factors. We found that candesartan markedly reduced the expression of gluconeogenic genes such as *G6pc* and *Pepck* in HFD-fed mice (Supplementary Fig. 8a), indicating that insulin suppresses hepatic glucose production. Moreover, candesartan dramatically reduced the expression of *Atf3* and *Xbp1* (Fig. 6e), both of which are key transcription factors for ER stress and the unfolded protein response (UPR)^9^, suggesting that candesartan normalized dysregulated ER stress and the UPR in HFD-fed mice. Furthermore, candesartan markedly reduced the expression levels of *Fabp4* and *Cd36*, which are genes involved in lipid uptake^49^, and *Cidea* and *Cidec*, which are genes involved in lipid droplet formation^50^ (Supplementary Fig. 8b, c), but did not change the expression of genes associated with lipid synthesis and β-oxidation, including *Acc1*, *Acc2*, *Fas*, and *Cpt1* (Supplementary Fig. 8d). These data suggest that the candesartan-mediated prevention of hepatic steatosis is likely driven by the suppression of genes associated with lipid uptake and lipid droplet formation. Indeed, candesartan-mediated attenuation of intracellular Ca^2+^ overload markedly reduced the expression levels of many transcription factors for lipid metabolism, including *Nur77*, *Nurr1*, *Pparδ*, and *Pparγ* (Fig. 6f). In line with these results, immunoblot analysis confirmed that candesartan significantly reduced the levels of NURR1, PPARγ, and XBP1 compared with those in control mice (Fig. 6g, h). These results suggest that the normalization of intracellular Ca^2+^ homeostasis is a crucial component of physiological and metabolic homeostasis.

### Candesartan ameliorates obesity-induced insulin resistance in HFD-fed mice by stimulating membrane localization of the AKT PH domain

**Fig. 7:**
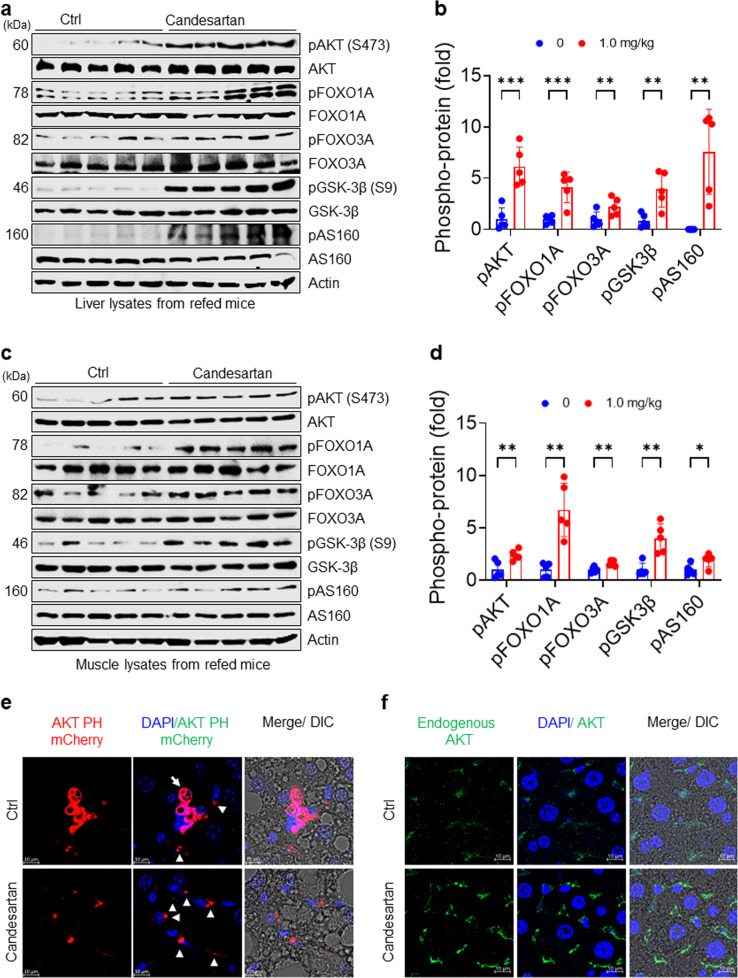
Candesartan enhances insulin signaling by stimulating postprandial membrane localization of AKT PH domains in HFD-fed mice. **a** Immunoblot analysis of liver extracts of mice after 16 h of overnight fasting and subsequent refeeding with HFD for 4 h. **b** The levels of phospho-proteins in (**a**) were quantified using ImageJ. **c** Immunoblot analysis of muscle extracts of mice after 16 h of overnight fasting and subsequent refeeding with HFD for 4 h. **d** The levels of phospho-proteins in (C) were quantified using ImageJ. **e** Representative fluorescence images of adenoviral Akt PH mCherry from mice administered vehicle or 1.0 mg/kg candesartan (scale bars: 5 μm). **f** Representative fluorescence images of endogenous AKT from mice administered vehicle or 1.0 mg/kg candesartan. Endogenous AKT-expressing ex vivo hepatocytes obtained from formalin-fixed liver sections of mice after 16 h of fasting and subsequent refeeding with HFD for 4 h were visualized using confocal microscopy (scale bars: 5 μm). All comparisons were conducted by two-way ANOVA with the Holm*-*Šídák’s post hoc test. All data are presented as the mean ± SD (*n* = 5 per group). *, *p* < 0.05; **, *p* < 0.01; ***, *p* < 0.001, ****, *p* < 0.0001, compared with controls.

To delineate the role of candesartan on insulin signaling in vivo, we measured postprandial phosphorylation of AKT and its downstream signaling substrates FOXO1A, FOXO3A, GSK-3β, and AS160 after fasting mice overnight and subsequently refeeding them a HFD for 4 h. In line with its in vitro effects, candesartan dramatically increased the postprandial phosphorylation of AKT and its downstream signaling molecules FOXO1A, FOXO3A, GSK-3β, and AS160 compared with that in control mice (Fig. 7a). Remarkably, quantification of immunoblots confirmed that postprandial phosphorylation of AKT and its downstream substrates in the liver tissues of candesartan-treated mice was fourfold to tenfold higher than that in control mice (Fig. 7b), suggesting that candesartan fully rescued impaired insulin signaling in HFD-fed mice. Furthermore, we confirmed that candesartan administration similarly improved postprandial insulin signaling in muscle tissues (Fig. 7c, d). These results demonstrate that the administration of candesartan, a drug that suppresses PA-induced intracellular Ca^2+^ overload, in HFD-fed mice substantially reversed obesity-induced insulin resistance by improving postprandial insulin signaling.

To investigate whether candesartan modulates membrane localization of the AKT PH domain, we examined the subcellular localization of the adenoviral AKT PH mCherry vector as a biosensor for PI(3,4,5)P_3_ in mice fed a HFD and treated with vehicle (control) or 1.0 mg/kg candesartan per day. The adenoviral AKT PH mCherry was mainly localized in the cytoplasm (white arrow in Fig. 7e), although some punctate adenoviral AKT PH mCherry became localized to the plasma membrane (arrowhead in Fig. 7e) in hepatocytes of HFD-fed mice in response to refeeding (Fig. 7e). These results are consistent with our previous finding that obesity-mediated Ca^2+^-PIPs prevent membrane localization of AKT PH domains in vivo^15^ and suggest that candesartan inhibits the formation of obesity-mediated Ca^2+^-PIPs, leading to improved insulin signaling.

Next, we monitored the subcellular localization of endogenous AKT in the livers of mice fed a HFD with vehicle or 1.0 mg/kg candesartan per day. In the hepatocytes of the mice fed a HFD without candesartan, endogenous AKT partially localized to the plasma membrane in response to refeeding (Fig. 7f), indicating that obesity-associated Ca^2+^-PIPs prevented the membrane localization of endogenous AKT. Surprisingly, in hepatocytes of mice on a HFD and treated with 1.0 mg/kg candesartan, endogenous AKT and IRS2 predominantly accumulated on the plasma membrane in response to refeeding (Fig. 7f and Supplementary Fig. 9), demonstrating that candesartan improves insulin sensitivity by facilitating postprandial membrane localization of PH domain-containing proteins such as AKT and IRS2. These results provide direct evidence that candesartan, a drug that inhibits obesity-associated intracellular Ca^2+^ overload, ameliorates obesity-induced insulin resistance by enhancing the postprandial membrane localization of AKT PH domains, leading to improved insulin sensitivity and signaling.

## Discussion

Insulin resistance is an impaired biological response to physiological insulin levels in tissues such as the liver, muscles, and adipocytes. Insulin resistance leads to metabolic syndromes, including hyperglycemia, hyperinsulinemia, hyperuricemia, dyslipidemia, low-grade inflammation, ER and mitochondrial dysfunction, and visceral adiposity^1–3,8–10^. There is mounting evidence that dysregulated intracellular Ca^2+^ homeostasis plays a crucial role in the pathogenesis of insulin resistance, obesity, diabetes, and diabetic cardiomyopathy^1,2,9,46,51,52^. Recently, we demonstrated that excess intracellular Ca^2+^ binds tightly with plasma membrane PIPs such as PI(3,4)P_2_, PI(4,5)P_2_, and PI(3,4,5)P_3_ to form Ca^2+^-PIPs under conditions of obesity-associated intracellular Ca^2+^ overload^15^. These Ca^2+^-PIPs prevent membrane targeting of essential PH domain-harboring proteins such as IRS1 and AKT, suggesting that excess intracellular Ca^2+^ causes impaired insulin signaling by interfering with electrostatic interactions between PH domains and PIPs^15^. It is still unclear, however, whether drugs that inhibit intracellular Ca^2+^ overload have beneficial effects on insulin resistance, and we have not yet been able to develop effective therapeutic strategies for correcting insulin resistance in obese individuals.

**Fig. 8:**
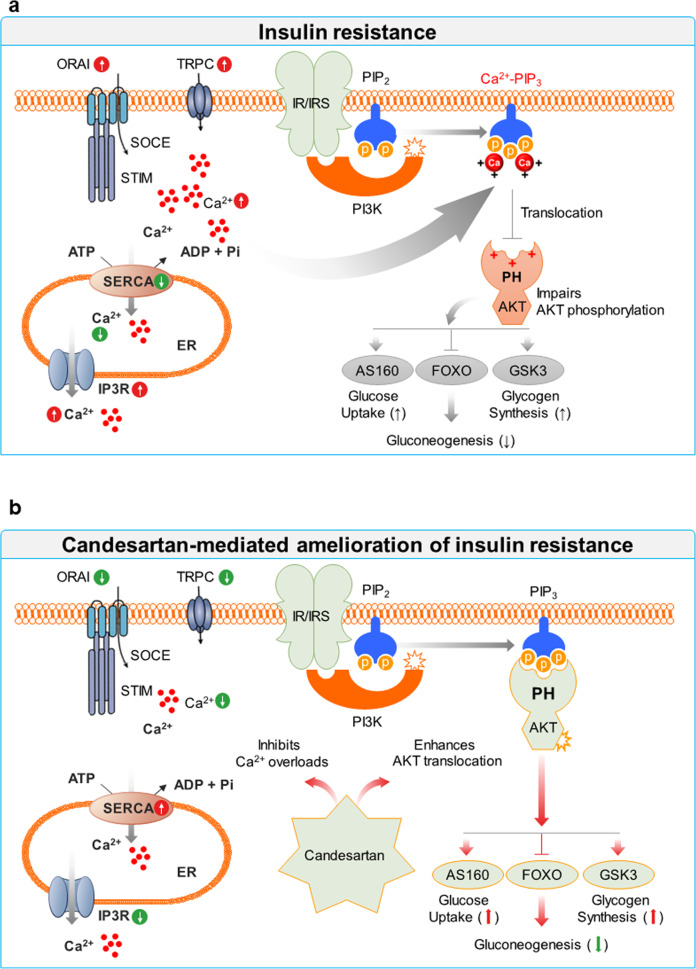
A proposed model of candesartan-mediated alleviation of insulin resistance in obesity. **a** Models show that metabolic stress could cause excess intracellular Ca^2+^ under pathophysiological conditions via SOC-mediated Ca^2+^ influx, whereby excess intracellular Ca^2+^ prevents the recognition of PIP3 by the AKT PH domain and impairs insulin signaling through the formation of Ca^2+^-PIP_3_. **b** In contrast, candesartan normalizes intracellular Ca^2+^ homeostasis in obesity by downregulating the expression of proteins associated with SOC-mediated Ca^2+^ influx, such as ORAI, STIM, and TRPC, or by upregulating SERCA levels. Thus, candesartan alleviates obesity-induced insulin resistance by enhancing the postprandial membrane localization of AKT PH domains, leading to improved insulin sensitivity and signaling.

Our study provides evidence that drugs that normalize intracellular Ca^2+^ homeostasis can ameliorate insulin resistance by rescuing impaired phosphorylation of AKT, an essential kinase for insulin signaling, and its downstream signaling molecules in the setting of obesity and hyperlipidemia. Under normal physiological conditions, balanced intracellular Ca^2+^ levels transduce the optimal kinetics of insulin signaling. Obesity or hyperlipidemia leads to excess intracellular Ca^2+^ levels through SOC-mediated Ca^2+^ influx, causing the formation of Ca^2+^-PIPs that inhibit the membrane interactions of PH domains, resulting in impaired insulin signaling. Thus, we demonstrate that candesartan ameliorates insulin resistance, hepatic steatosis, and tissue inflammation in obesity by normalizing dysregulated intracellular Ca^2+^ homeostasis (Fig. 8).

Following the pathophysiological concept that excess intracellular Ca^2+^ acts as a critical negative regulator of insulin signaling, we screened nine ARBs that are approved by the FDA to treat hypertension and found that azilsartan and candesartan improved insulin signaling by suppressing obesity-associated intracellular Ca^2+^ overload in the setting of obesity. We first showed that candesartan and azilsartan could alleviate insulin resistance in PA-treated HepG2 cells by fully restoring the impaired insulin-stimulated phosphorylation of AKT and its downstream substrates GSK3β, AS160, FOXO1, and FOXO3. Because other ARBs did not have the same effects, these results suggest that the effects of candesartan and azilsartan on insulin signaling are outside the scope of the action of ARBs as AT1R antagonists^27,53^. Given that the renin-angiotensin system is frequently activated in patients with nonalcoholic fatty liver disease (NAFLD) and that candesartan significantly attenuates liver fibrosis in obese mice and type 2 diabetes^23,31^, our findings suggest that candesartan suppresses macrophage infiltration and hepatic inflammation by normalizing intracellular Ca^2+^ homeostasis. Further work is required to investigate the effect of candesartan on hepatic fibrosis and the activation of hepatic stellate cells associated with dysregulated intracellular Ca^2+^ homeostasis in obesity.

We hypothesized that candesartan and azilsartan rescued impaired insulin signaling by modulating intracellular Ca^2+^ levels because our previous results showed that excess intracellular Ca^2+^ negatively affects insulin signaling^15^. This hypothesis was supported by our findings that candesartan and azilsartan, but not the other ARBs, dramatically suppressed PA-induced intracellular Ca^2+^ overload in HepG2 cells. Furthermore, Ca^2+^ imaging analysis suggested that candesartan reduced sustained Ca^2+^ overload by inhibiting dysregulated SOCE in PA-treated HepG2 cells. Indeed, qRT‒PCR and immunoblot analyses indicated that the candesartan-mediated suppression of PA-induced intracellular Ca^2+^ overload in HepG2 cells was driven by reduced expression of SOCE-associated proteins such as ORAI2, STIM1, TRPC1, TRPM4, and TRPM7^9,54,55^ and enhanced expression of SERCA2, which is consistent with previous findings that the insulin-sensitizing agent rosiglitazone upregulated SERCA expression^9,56^. Concurrent with the decrease in intracellular Ca^2+^ levels, candesartan and azilsartan fully rescued impaired insulin-stimulated membrane localization of the AKT PH domain in PA-treated HepG2 cells, thereby improving insulin sensitivity and signaling. These results imply that specific inhibition of PA-induced intracellular Ca^2+^ overload might be an effective therapeutic strategy to ameliorate insulin resistance. Importantly, an increased intracellular Ca^2+^ concentration induces programmed cell death via inactivation of Akt^9,57^. Akt regulates^58^ cell death by phosphorylating and inactivating components of the apoptotic machinery, including BAD and Caspase-9.

In addition to our in vitro results in PA-treated HepG2 cells, we showed that candesartan improved metabolic profiles such as glucose tolerance, insulin sensitivity, and the HOMA-IR index in HFD-fed mice. The reduced HOMA-IR index, indicating lower insulin resistance, in candesartan-treated mice was mainly due to reduced serum insulin levels, which is in agreement with previous studies showing that candesartan improved insulin sensitivity in obese rats^59^ and patients with essential hypertension^24,60^. This candesartan-mediated enhancement of insulin sensitivity resulted in improved hepatic function, reduced hepatic steatosis and hyperlipidemia, and reduced adipocyte inflammation in HFD-fed mice. Furthermore, consistent with our in vitro results, candesartan treatment in HFD-fed mice increased the expression of SERCA2 and reduced the expression of the SOCE-associated proteins ORAI2, STIM1, TRPC1, TRPM4, and TRPM7, suggesting that candesartan modulates intracellular Ca^2+^ homeostasis in vivo. Finally, we found that candesartan enhanced postprandial membrane localization of AKT PH domains, resulting in increased phosphorylation of AKT and its downstream substrates FOXO1A, FOXO3A, GSK3β, and AS160. Taken together, these results suggest that candesartan-mediated normalization of intracellular Ca^2+^ homeostasis may translate into enhanced insulin signaling and improved systemic metabolism and overall metabolic health in the setting of obesity.

Drug repurposing is a valuable strategy in which approved drugs are used in therapeutic applications other than those for which they were originally designed. ARBs are antihypertensive drugs that provide additional benefits, including a reduction in diabetes risk, enhancement of insulin sensitivity, and reduction in microalbuminuria^23,61^. We screened nine different ARBs and found that candesartan and azilsartan could alleviate impaired insulin signaling in PA-treated HepG2 cells, whereas the other ARBs did not show any effects on insulin signaling. Furthermore, candesartan and azilsartan markedly reduced the intracellular Ca^2+^ concentrations and rescued insulin-stimulated membrane localization of AKT PH domains in PA-treated HepG2 cells. The chemical structures of candesartan and azilsartan are nearly identical^53^, except that a tetrazole ring in candesartan is replaced by a 5-oxo-1,2,4-oxadiazole ring in azilsartan. This chemical similarity might explain the similar pharmacological effects of candesartan and azilsartan on insulin resistance by reducing intracellular Ca^2+^ overload and lipid accumulation, whereas other ARBs did not have any effects on reducing intracellular Ca^2+^ overload and lipid accumulation (Supplementary Fig. 9). Additional experiments are required to identify the relevant direct therapeutic targets of candesartan and azilsartan in the setting of PA-induced intracellular Ca^2+^ overload.

## Supplementary information

Supplementary Materials
