# Supplementary material for: Candesartan, an angiotensin-II receptor blocker, ameliorates insulin resistance and hepatosteatosis by reducing intracellular calcium overload and lipid accumulation
Source: Exp Mol Med. 2023 May 1;55(5):910–25. doi: 10.1038/s12276-023-00982-6 (PMC10238434; doi:10.1038/s12276-023-00982-6)
Supplement: Supplementary file 1 — Supplementary Materials [file 12276_2023_982_MOESM1_ESM.pdf]

Candesartan, an angiotensin-II receptor blocker, ameliorates insulin resistance and hepatosteatosis by reducing intracellular calcium overload and lipid accumulation

Jin Wook Lee[1,2], Hyun-Oh Gu[1,2], Yunshin Jung[2], YunJae Jung[2,3], Seung-Yong Seo[4], Jeong-Hee Hong[1], In-Sun Hong[5], Dae Ho Lee[6], Ok-Hee Kim[1]*, and Byung-Chul Oh[1,2*]

[1]Department of Physiology, Lee Gil Ya Cancer and Diabetes Institute, Gachon College of Medicine, Incheon, 21999, Republic of Korea

[2]Department of Health Sciences and Technology (GAIHST), Gachon University, Incheon 21999, Korea,

[3]Department of Microbiology, Lee Gil Ya Cancer and Diabetes Institute, Gachon University College of Medicine, Incheon, 21999, Republic of Korea

[4]College of Pharmacy, Gachon University, Incheon, 21936, Republic of Korea

[5]Department of Molecular Medicine, Lee Gil Ya Cancer and Diabetes Institute, Gachon University College of Medicine, Incheon, 21999, Republic of Korea

[6]Department of Internal Medicine, Gachon University Gil Medical Center, Incheon, 21565, Republic of Korea

Corresponding authors:

Address correspondence to Byung-Chul Oh (bcoh@gachon.ac.kr) and (ohkim323@gmail.com)

Supplementary Materials and Methods

Glucose tolerance test and insulin tolerance test

IP-GTT was performed by intraperitoneal injection of glucose (1 g /kg body weight) after a 16 h fast. Blood was collected from a small incision at the tip of the tail at 0, 15, 30, 60, and 90 min after glucose injection. Blood glucose levels were measured with a blood glucometer (Accu-Check Aviva, Roche). IP-ITT was performed by intraperitoneal injection of insulin (0.75 U/kg body weight; Actrapid Penfill, Novo Nordisk) after a 4 h fast. Blood glucose levels were measured at 0, 15, 30, 60, and 120 min after insulin injection. HOMA-IR was calculated using the following formula: (fasting insulin × fasting glucose)/405.

Determination of body composition and liver fat content

The body composition of mice was measured using a nuclear magnetic resonance imaging technique (Minispec Live Mice Analyzer (LF50), Bruker) at the end of the experiments. Frozen liver tissues were homogenized in modified RIPA buffer. Triglyceride contents and total proteins in liver lysates were measured with AU480 biochemistry. Triglyceride contents were normalized by the total protein concentration.

Serum and urine biochemical assay

At the end of the experiments, the mice were fasted for 16 h and refed with an HFD before receiving isoflurane. Abdominal aorta blood was obtained. Liver, kidney, and adipose tissues were dissected and stored at −80°C. Serum biochemical analyses of ALT, AST, TG, total cholesterol, LDL-cholesterol, and HDL-cholesterol were performed as described previously using a Model AU-480 (Beckman Coulter, Fullerton, CA, USA).

Monitoring of subcellular localization of AKT PH domain

CHO cells or HepG2 cells were transfected with cDNA encoding hAKT PHD (residues 1–144) using Lipofectamine (Life Technologies) as described previously [1]. To monitor the subcellular localization of endogenous AKT, we subjected liver sections to antigen retrieval with 10 mM Tris/HCl (pH 9.0) for 20 min, followed by treatment with serum-free protein blocking solution (DAKO) for 30 min and permeabilization with 0.5% Tween 20 for

15 min. Then, we incubated the liver sections with an anti-AKT PHD antibody (SKB1, #05-591, Sigma-Aldrich) followed by a FITC-conjugated secondary antibody. After mounting, the liver sections were imaged with an LSM 980 laser-scanning confocal microscope (Carl Zeiss). Images were analyzed with ZEN Software (Carl Zeiss).

The AKT PH mCherry adenovirus (5×10^8 plaque-forming units) was delivered by systemic tail-vein injection into C57BL/6J mice after 8 weeks of HFD feeding and 2 weeks of treatment with 1.0 mg/kg candesartan or vehicle, as described previously [2]. Seven days after adenoviral infection, the livers were collected from the mice following an overnight fast and subsequent refeeding with HFD for 4 h. For immunofluorescence, formalin-fixed liver sections or cells were plated onto slide glass and counterstained with DAPI to visualize the nuclei. After mounting, the sections were imaged with a Zeiss LSM 700 laser-scanning confocal microscope (Carl Zeiss) and analyzed with ZEN Software (Carl Zeiss). Intracellular Ca^{2+} was quantified using NIH ImageJ software (https://imagej.nih.gov/ij/, 1997-2017). Data are presented as fold changes.

Measurement of intracellular Ca^{2+} concentration and lipid accumulation

For intracellular Ca^{2+} concentration and lipid accumulation, HepG2 cells were plated onto slide glass; treated with 0.5 mM PA for 16 h; incubated with 4 μM Fura-2-AM (Teflabs), 5 μM Calbryte 630 AM, or 2 μM of BODIPY for 30 min in PBS at 37°C in the dark; and then washed for 10 min with PBS. Then cells were counterstained with 4,6-diamidino-2-phenylindole (DAPI) to visualize the nuclei. After mounting, the stained slides were imaged with a Zeiss LSM 700 or Zeiss LSM 980 laser-scanning confocal microscope (Carl Zeiss) and analyzed with ZEN 2010 Software (Carl Zeiss). Intracellular Ca^{2+} was quantified from the microscopy images using NIH ImageJ software (https://imagej.nih.gov/ij/, 1997-2017). Data are presented as fold changes.

Measurement of intracellular Ca^{2+} concentration ($[Ca^{2+}]_i$)

The $[Ca^{2+}]_i$ signaling was measured using fura-2 AM, a fluorescent dye for cytosolic Ca^{2+}. Cells were attached to coverslips and incubated with 4 μM fura-2 AM and 0.05% pluronic acid for 15 min at room temperature in the dark. The dye-loaded cells were then placed on an inverted microscope and perfused with physiological salt solution solutions to detect

$[Ca^{2+}]_i$. The $[Ca^{2+}]_i$ was determined by the fura-2 ratio of dual excitation wavelengths 340 nm and 380 nm (emission wavelength is 530 nm). The emitted fluorescence was monitored by a CCD camera (Q-imaging) attached to an inverted microscope (Nikon, Japan) and analyzed with a Metafluor system (Molecular Devices, USA). For measurement of Ca^{2+} influx, cells were stimulated with the sarco/endoplasmic reticulum Ca^{2+} ATPase inhibitor cyclopiazonic acid (CPA) in Ca^{2+}-free solution to deplete Ca^{2+} stores and then administered 5 mM Ca^{2+}-containing PBS.

RNA sequencing and data analysis

Total RNA was isolated from liver tissues using the RNAiso Plus reagent (Takara, Shiga, Japan), and genomic DNA was removed by treatment with RNase-free DNase (Roche, Manheim, Germany). The concentration and integrity of the RNA in each sample were measured on a 2100 Bioanalyzer (Agilent) with an RNA integrity threshold value ≥ 8. A TruSeq RNA library kit (Illumina, San Diego, CA, USA) was used to prepare cDNA libraries. The resulting libraries were amplified by 15 cycles of PCR and then sequenced by Macrogen (Seoul, Korea) using a HiSeq™ 2000 sequencing system (Illumina) to produce 100-bp paired-end reads. Differentially expressed genes were analyzed using the Fisher's exact test, and multiplicity correction was performed using the Benjamini–Hochberg method on the resulting *p* values to control the false discovery rate (FDR). The exact p value and FDR q value resulting from the differential gene expression analysis can be found in the Source Data. Differentially regulated genes with an FDR value <0.05 were considered significant. We defined expressed genes as those with detectable expression in at least half of all samples. Expressed genes were subjected to principal component analysis (PCA). Principal components 1 and 2 were plotted in 2-D coordinates. Deposited Gene Expression Omnibus (GEO) RNA-seq genomic data are publicly available and contained in the Source Data. Moreover, we analyzed the enriched signaling pathways based on the differentially expressed genes identified using the Ingenuity Pathway Analysis software (IPA, QIAGEN). For the analysis, we removed the gene expressions with (a) log2-fold-change (log2FC) values between −1 and 1; (b) $p > 0.05$; (c) False Discovery Rate (FDR) > 0.05, and (d) FPKM < 1 for all samples.

qRT-PCR analyses

Total RNA was extracted from HepG2 cells and frozen liver, kidney, and adipose tissues with Trizol (Invitrogen) according to the manufacturer's protocol. RNA concentrations were measured on a Nanodrop spectrophotometer (Thermo Scientific). cDNA synthesis and real-time RT-PCR were performed using the TB Green Premix Ex Taq cDNA Synthesis kit (TaKaRa Bio, Tokyo, Japan) with 1 μg total RNA plus random hexamers in a BioRad CFX384. All expression values were normalized to the cyclophilin A mRNA level. Primer sequences are listed in **Supplementary Table 2 and 3**.

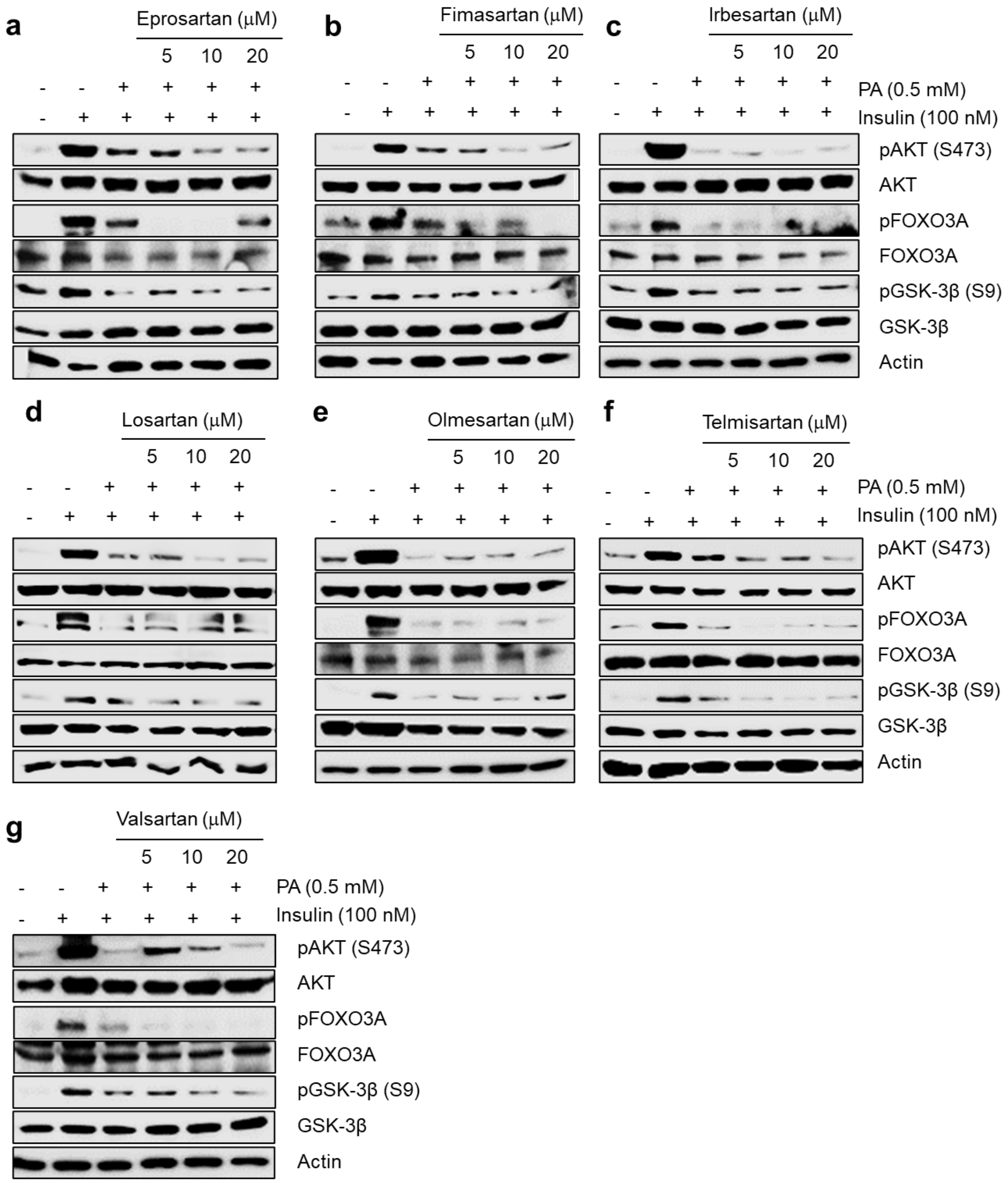

Supplementary Fig. 1. The effects of ARBs on insulin signaling in PA-treated HepG2 cells. (**a-g**) Immunoblot analysis of the human HepG2 cell lysates treated with 0.5 mM PA and the indicated concentrations of ARBs for 16 h followed by treatment with 100 nM insulin for 15 min.

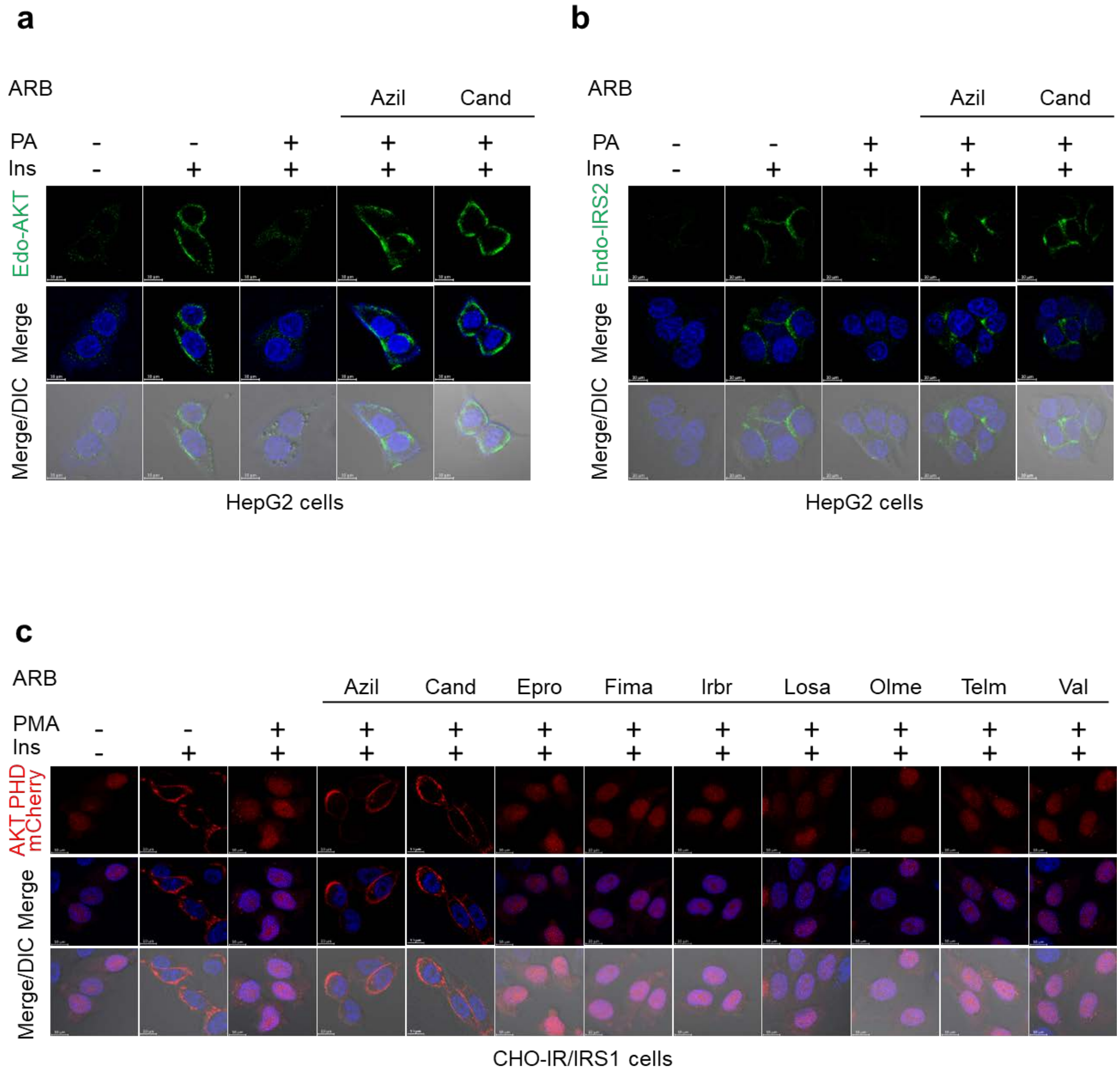

Supplementary Fig. 2. The effects of ARBs on membrane localization of the Akt PH domain, endogenous AKT, and endogenous IRS2. (**a**) Representative fluorescence images of endogenous AKT in HepG2 cells. (**b**) Representative fluorescence images of endogenous IRS2 in HepG2 cells. HepG2 cells were treated with 0.5 mM PA and the indicated ARBs for 16 h, serum-starved for 3 h, stimulated with 100 nM insulin for 15 min, counterstained with 4,6-diamidino-2-phenylindole (DAPI) for nuclei, and observed using differential interference contrast (DIC) microscopy. (**c**) Representative fluorescence images of CHO-IR cells harboring Akt PH domain-mCherry fusion vector. CHO-IR cells were transfected with Akt PH domain-mCherry fusion vector, treated with 100 nM PMA or 100 nM PMA and the indicated ARBs for 16 h, serum-starved for 3 h, stimulated with 100

nM insulin for 15 min, counterstained with 4,6-diamidino-2-phenylindole (DAPI) for nuclei, and observed using differential interference contrast (DIC) microscopy.

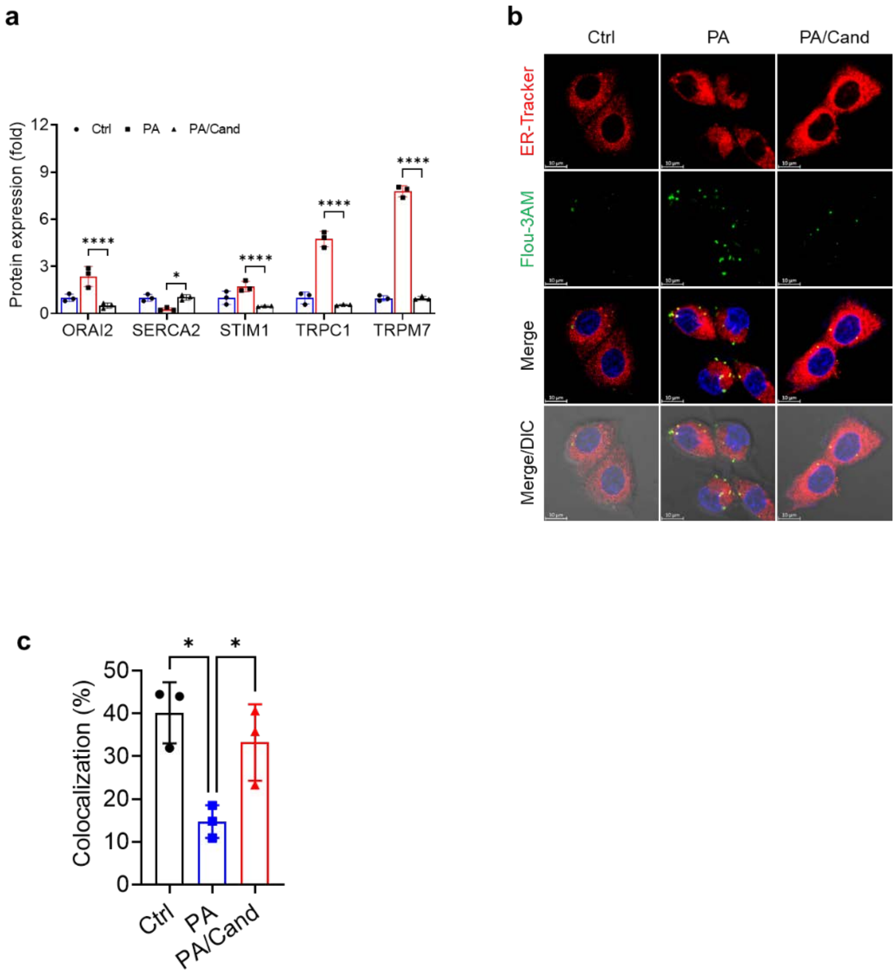

Supplementary Fig. 3. Candesartan normalizes dysregulated intracellular Ca^{2+} homeostasis by inhibiting store-operated Ca^{2+} entry (SOCE). (a) Quantification of the protein immunoblots in Figure 3H using ImageJ. All data are presented as the mean ± SD (n = 4–6 per group). *, p<0.05; **, p<0.01; ***, p<0.001, ****, p<0.0001, compared with controls. (**b**) Representative Fluo-3 AM images of cytosolic Ca^{2+} and ER Tracker images of the endoplasmic reticulum (ER) in HepG2 cells treated with vehicle, 0.5 mM PA, or 0.5 mM PA and 10 μM candesartan. HepG2 cells were incubated with the cytosolic Ca^{2+} indicator Fluo-3 AM (4 μM) and ER Tracker (1 μM) for 45 min, and visualized by confocal microscopy (scale bar 10 μm). Fluo-3 AM localized to the cytoplasm in a punctate distribution, thereby reflecting intracellular free Ca^{2+}. (**c**) Colocalization between Mag-Fluo-3 AM and ER tracker in HepG2 cells treated with vehicle, 0.5 mM PA, or 0.5 mM PA and 10 μM candesartan. All data are presented as the mean ± standard deviation (SD) (n = 3 per group). *, p<0.05; **, p<0.01; ***, p<0.001, ****, p<0.0001, compared with controls.

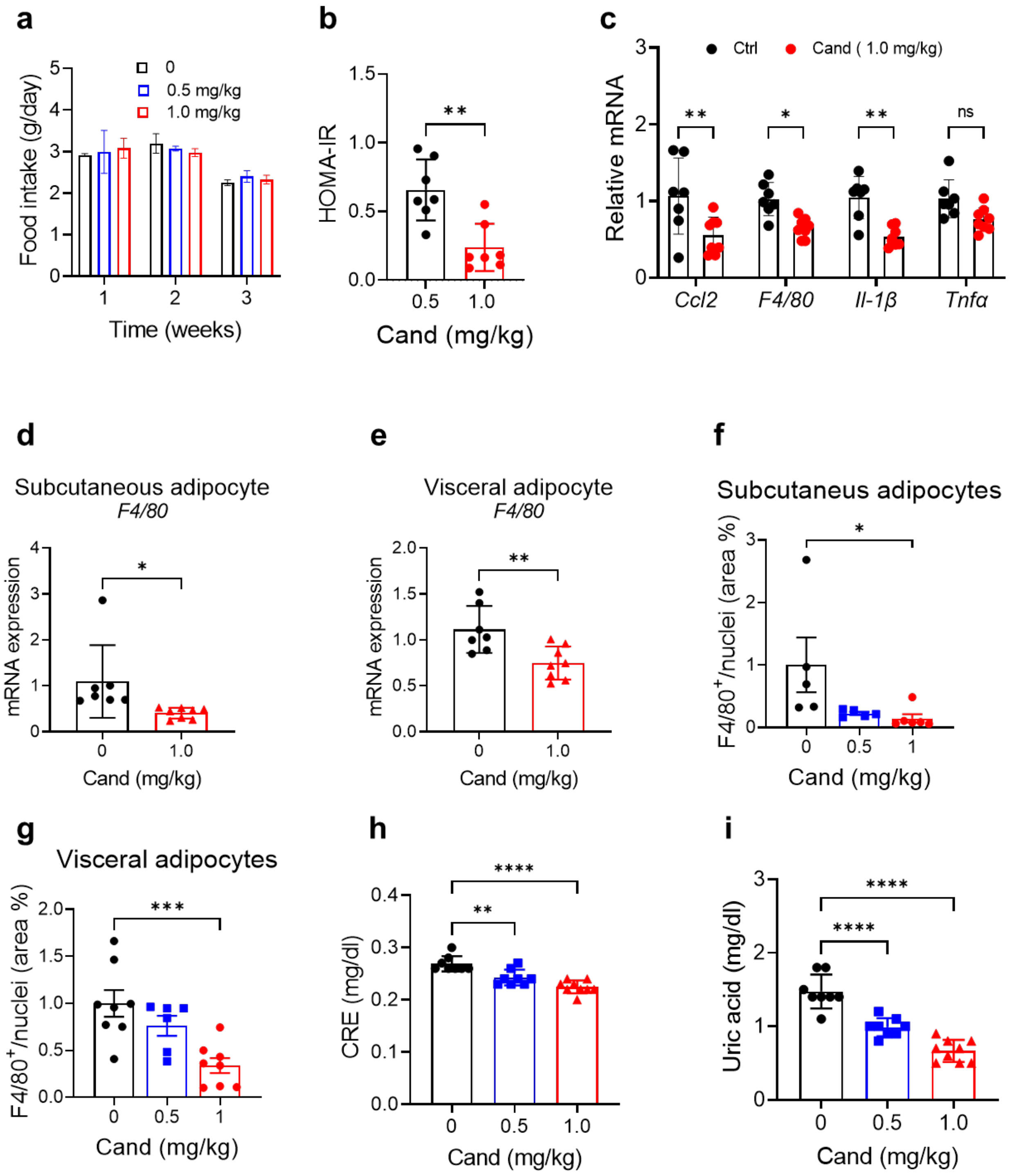

Supplementary Fig. 4. Candesartan alleviates insulin resistance and tissue inflammation. (a) Food intake of the mice during candesartan administration. (**b**) HOMA-IR index. The reciprocal HOMA-IR was calculated as (fasting glucose × fasting insulin)/405. (**c**) Relative mRNA expression of *Cccl2*, *F4/80*, *Il-1β*, and *Tnfα* in the livers

of mice fed a 60% HFD with vehicle or 0.5 mg/kg or 1.0 mg/kg candesartan. (**d**) Relative mRNA expression of *F4/80* in subcutaneous adipocytes of mice fed a 60% HFD with vehicle, or 0.5 mg/kg or 1.0 mg/kg candesartan. (**e**) Relative mRNA expression of *F4/80* in visceral adipocytes of mice fed a 60% HFD with vehicle or 0.5 mg/kg or 1.0 mg/kg candesartan. (**f**) The F4/80-positive area of subcutaneous adipocyte sections was quantified using Image J software. (**g**) The F4/80-positive area of visceral adipocyte sections was quantified using Image J software. (**h**,**i**) Serum levels of CRE (**h**), and Uric acid (**i**) were measured with a Beckman Coulter AU480 chemistry analyzer.

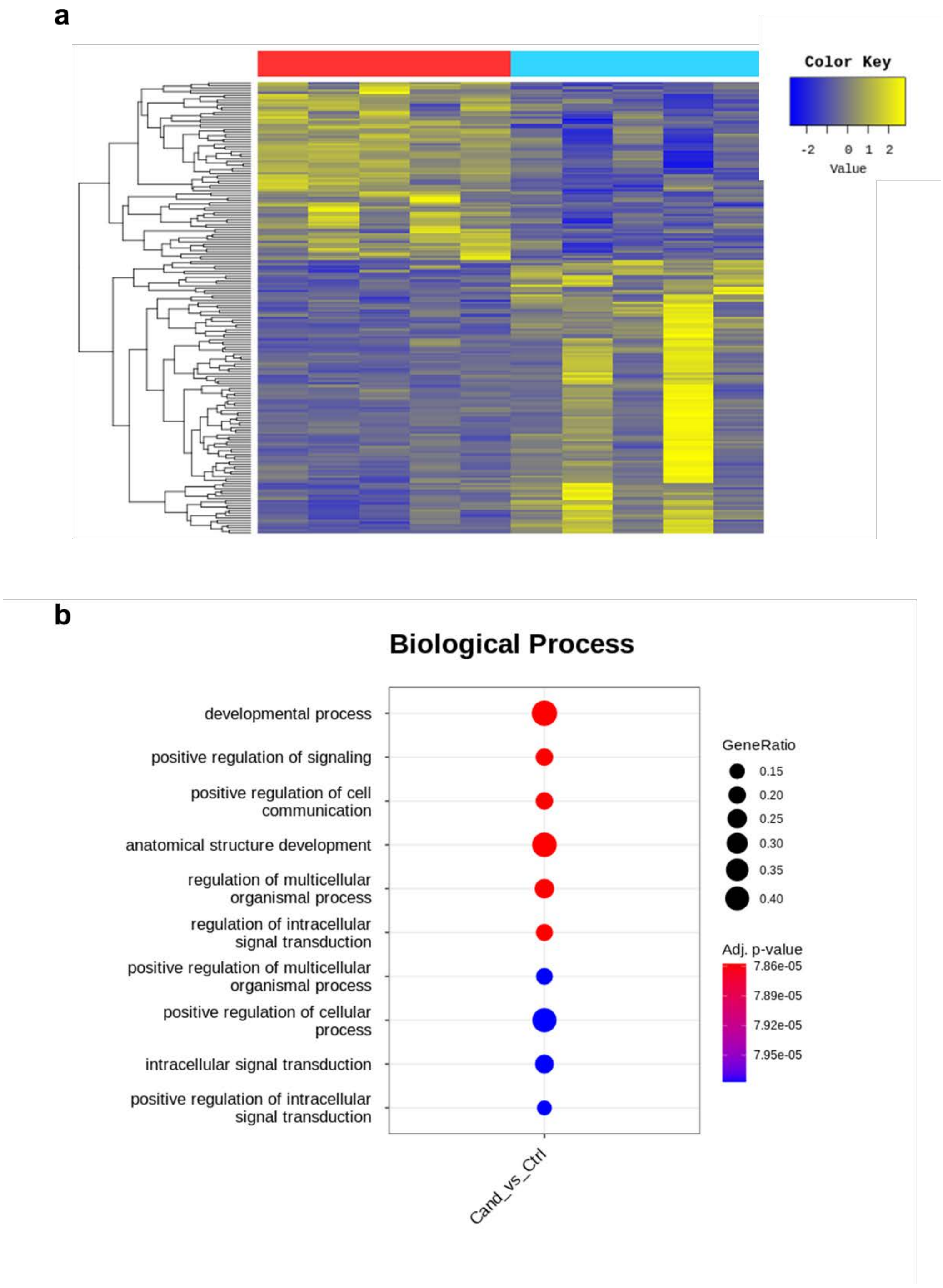

Supplementary Fig. 5. Candesartan alters the hepatic metabolism transcriptome. (**a**) Hierarchical clustering heatmap of differentially expressed genes. (**b**) Gene set enrichment analysis (GSEA) with gene ontology gene-sets reveals molecular pathways related to the positive regulation of signaling pathways and intracellular signaling.

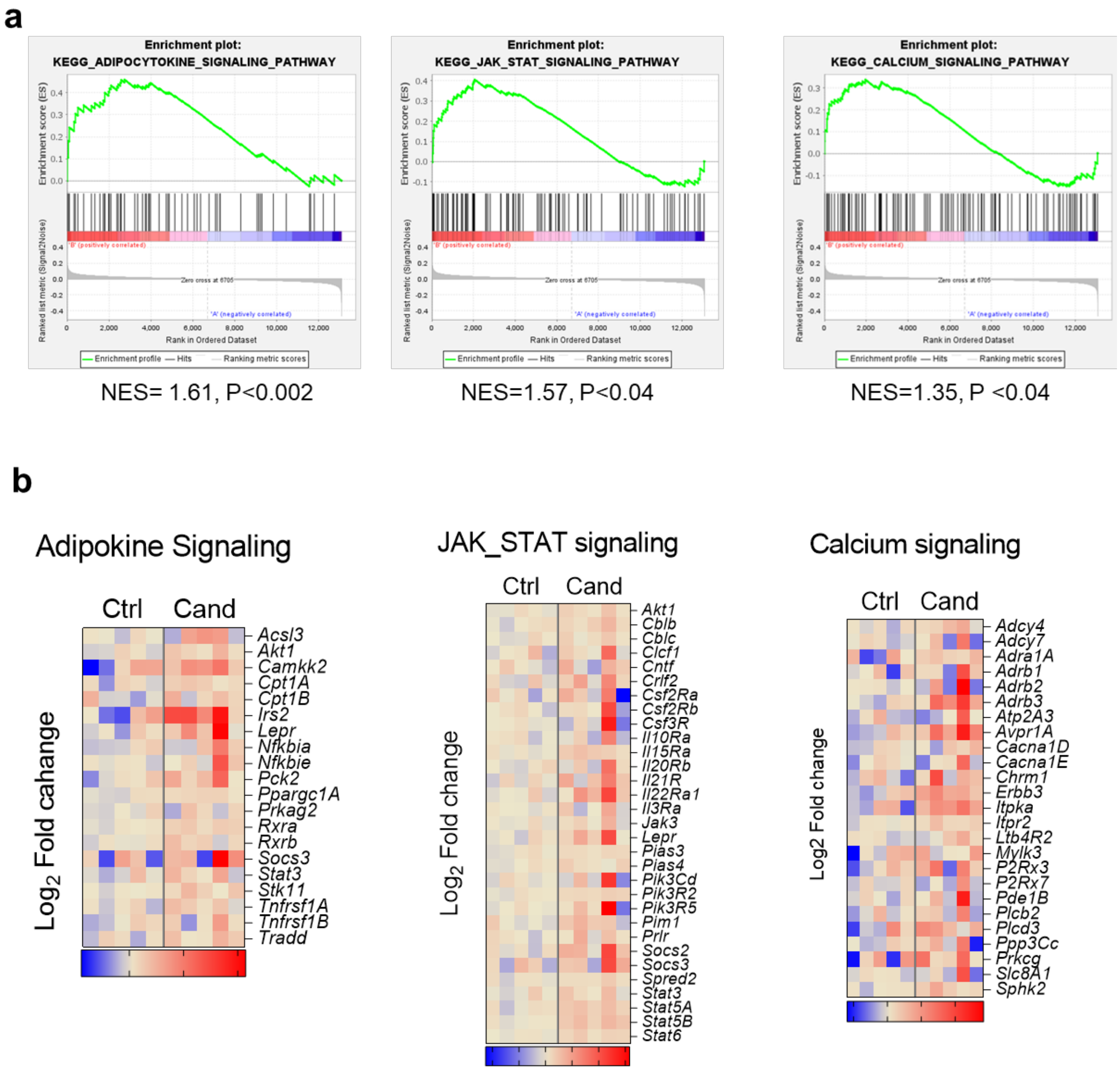

Supplementary Fig. 6. Candesartan alters the hepatic metabolism transcriptome. (**a**) Enrichment plot showing the upregulation of the adipokine signaling pathway, JAK-STAT pathway, and calcium signaling. (**b**) Functional clustering heatmap of differentially expressed genes associated with adipokine signaling, JAK-STAT signaling, and calcium signaling.

a

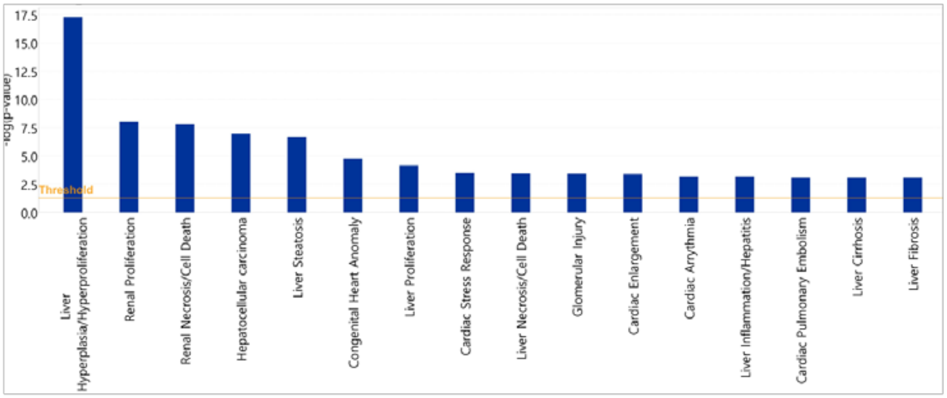

b

Categories	Diseases or Functions Annotation	p-value	Predicted Activation State	Activation z-score
Liver Hyperplasia/Hyperproliferation	Liver cancer	5.31E-18	Decreased	-2.267
Liver Hyperplasia/Hyperproliferation	Liver carcinoma	1.27E-17	Decreased	-2.645
Liver Hyperplasia/Hyperproliferation	Liver tumor	8.39E-17	Decreased	-2.589
Liver Inflammation/Hepatitis,Liver Steatosis	Steatohepatitis	0.00617	Decreased	-2.219

c

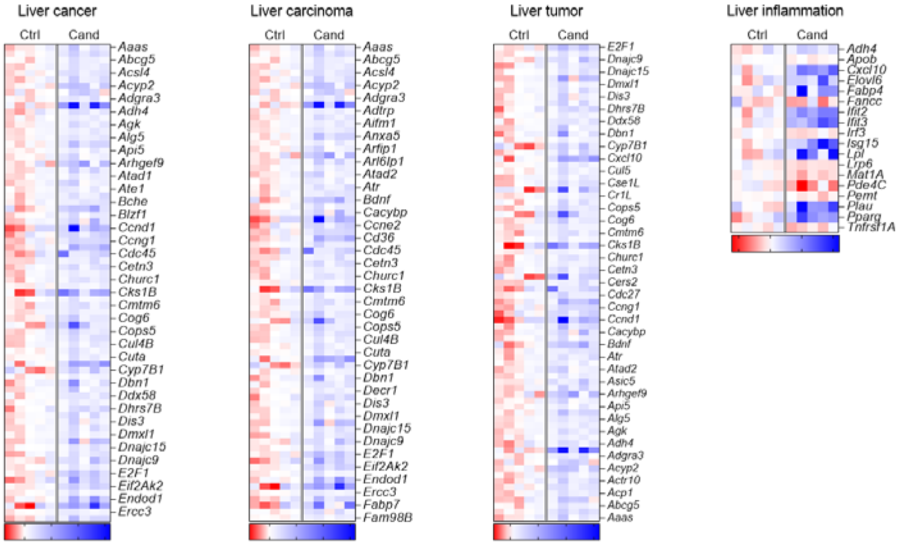

Supplementary Fig. 7. IPA analysis of transcriptome data indicates changes in several hepatotoxicity-related pathways after treating mice fed a HFD with 1 mg/kg candesartan including hyperplasia, steatosis, and carcinoma. (**a,b**) The most prominently affected pathways (“Tox Functions”) are depicted, as identified using the IPA software. The yellow line represents a value of −log (0.05), indicating that all bars above are statistically significant. (**c**) Functional clustering heatmap of differentially expressed genes associated with liver cancer, liver carcinoma, liver tumor, and liver inflammation.

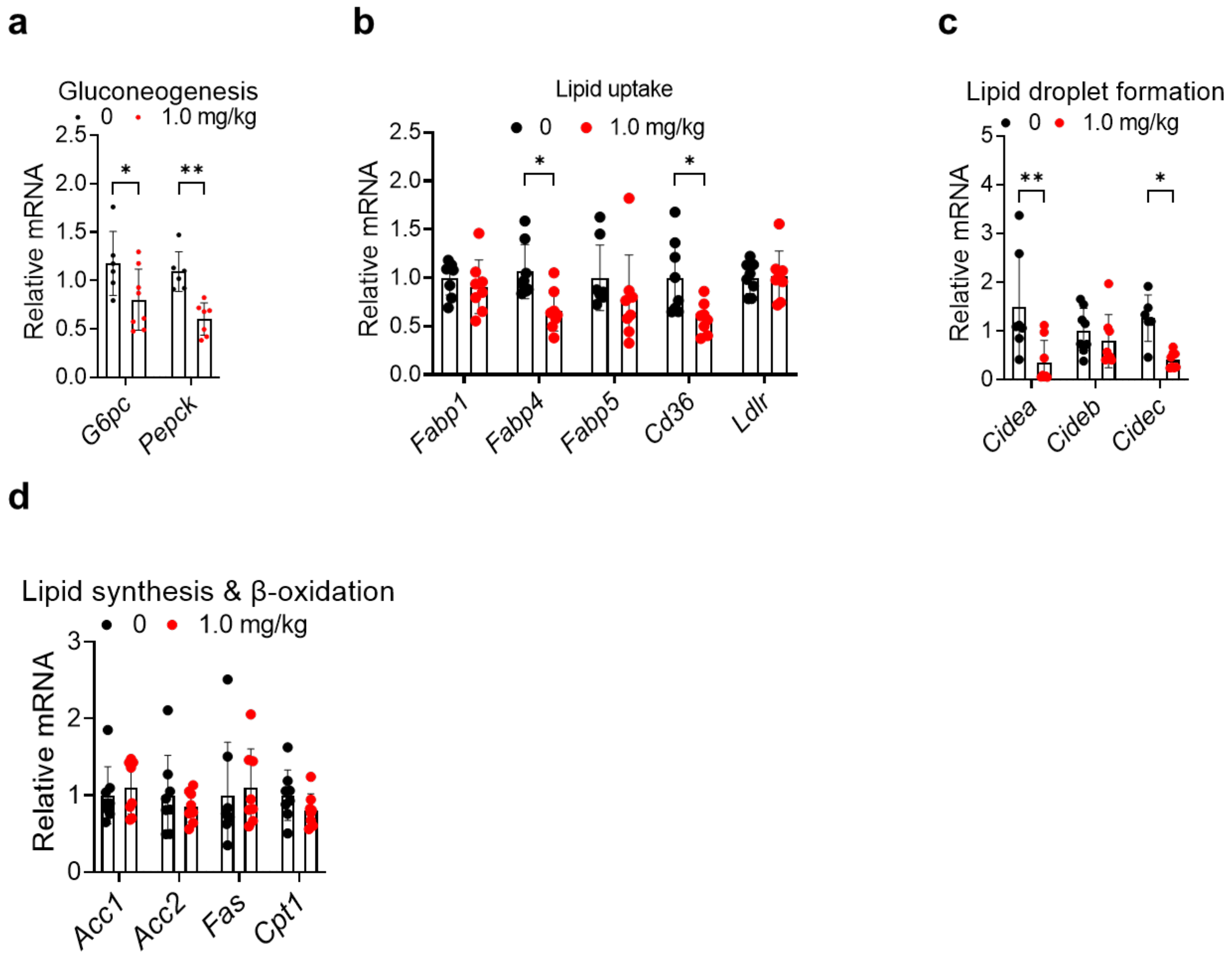

Supplementary Fig. 8. Candesartan suppresses the expression of genes associated with gluconeogenesis and lipid metabolism. (**a–d**) Relative mRNA expression of genes associated with gluconeogenesis (**a**), lipid uptake (**b**), lipid droplet formation (**c**), and lipid synthesis and β-oxidation (**d**) in the livers of mice fed a 60% HFD with vehicle or 1.0 mg/kg candesartan.

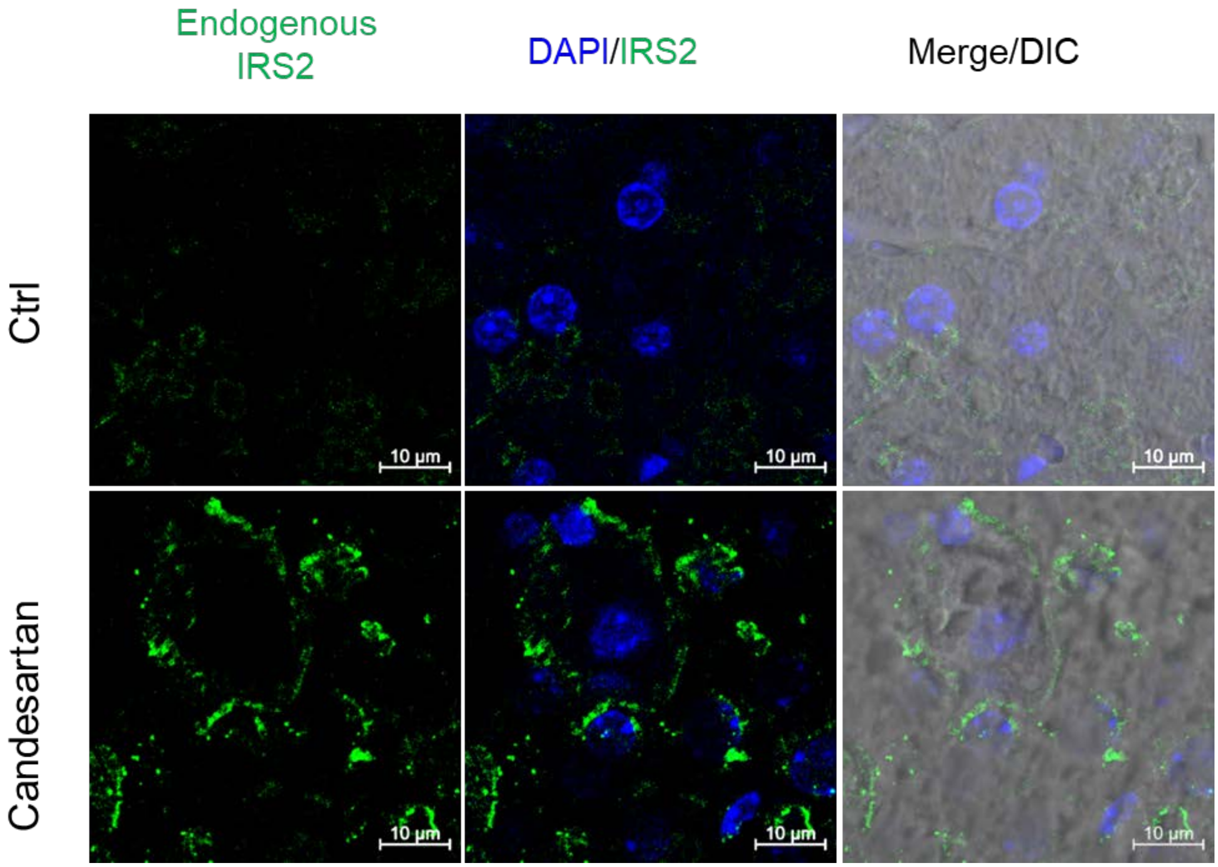

Supplementary Fig. 9. **Candesartan enhances insulin signaling by stimulating postprandial membrane localization of endogenous IRS2 in HFD-fed mice**. Representative fluorescence images of endogenous IRS2 from mice administered vehicle or 1.0 mg/kg candesartan. Endogenous IRS2-expressing *ex vivo* hepatocytes from formalin-fixed liver sections of mice after 16 h of fasting and subsequent refeeding with a HFD for 4 h were visualized by confocal microscopy (scale bars 10 μm).

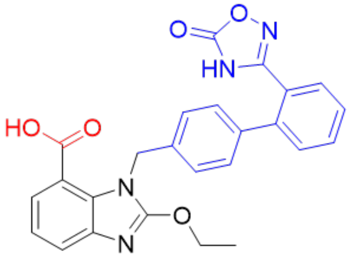

azilsartan

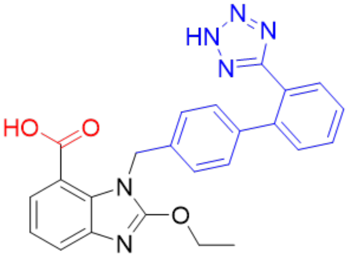

candesartan

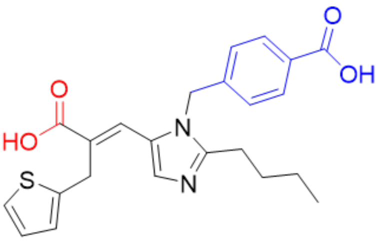

eprosartan

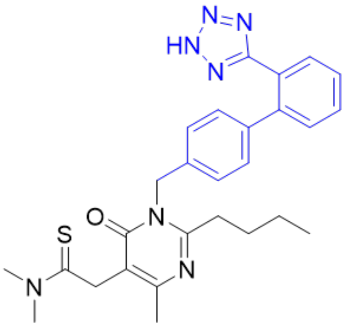

Fimasartan

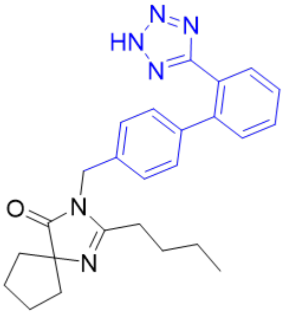

irbesartan

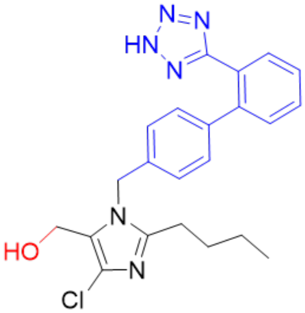

losartan

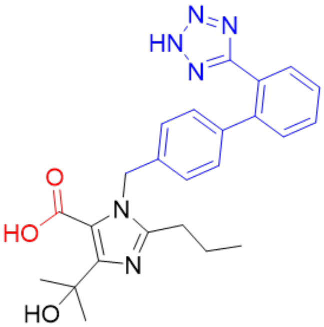

olmesartan

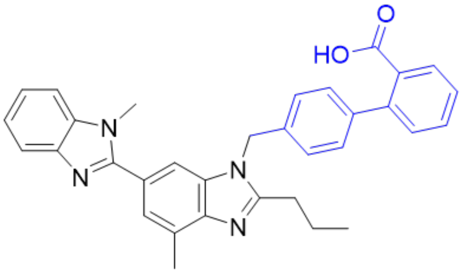

telmisartan

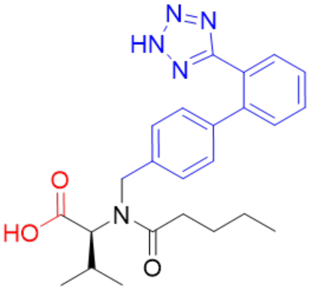

valsartan

Supplementary Fig. 10. **Chemical structures of angiotensin II receptor blockers (ARBs)**.

Supplementary Table 1. List of antibody, source, application and dilution

Antibody	Catalog Number	Application	Dilution
Rabbit anti-AKT	Cell signaling, C67E7, #4691	WB	1:5000
Rabbit anti-phospho-AKT S473	Cell signaling, D9E, #4060	WB	1:3000
Rabbit GSK-3β	Cell signaling, (27C10), #9315	WB	1:3000
Rabbit Phospho-GSK-3β S9	Cell signaling, #9336	WB	1:3000
Rabbit AS160	Cell signaling, #2670	WB	1:3000
Rabbit anti-phospho-AS160 Thr642	Cell signaling, D27E6 #8881	WB	1:3000
Rabbit STIM2	Cell signaling, #4917	WB	1:3000
Rabbit Anti-FOXO1A	Abcam, ab52857	WB	1:1000
Rabbit Anti-phospho-FOXO1A S253	Abcam, ab259337	WB	1:1000
Rabbit Anti-NUR77 antibody	Abcam, ab153914	WB	1:1000
Rabbit Anti-PPAR alpha	Abcam, ab215270	WB	1:1000
Rabbit Anti-PPAR gamma	Abcam, ab209350	WB	1:1000
Rabbit Anti-Stromal interaction molecule 1 (STIM1)	Abcam, ab108994	WB	1:3000
Rabbit Anti-SERCA2 ATPase	Abcam, ab150435	WB	1:1000
Rabbit Anti-TRPC1	Abcam, ab192031	WB	1:3000
Rabbit Anti-TRPM7	Abcam, ab245408	WB	1:500
Rabbit Anti-FOXO3a/ FKHRL1	Millipore, 07-702	WB	1:1000
Rabbit Anti-phospho-FKHRL1/FOXO3A Thr32	Millipore, 07-695	WB	1:1000
Goat ITPR2	Novusbio, NB100-2466	WB	1:1000
Rabbit ORAI2	Proteintech, 20592-1-AP	WB	1:1000
Rabbit anti-ATP2B2 / PMCA2	LS-bio, LS-C348973	WB	1:1000
Rabbit anti-TRPM4	LS-Bio, LS-C408114	WB	1:1000
Rabbit CD36	Bioss, bs-8873	WB	1:1000
Mouse Nurr1	Santa cruz, sc-376984	WB	1:500
Rabbit XBP1	Santa cruz, sc-7160	WB	1:500
Mouse Anti-β-Actin, clone C4	Millipore, MAB1501	WB	1:10000
Rat F4/80 Monoclonal	eBioscience™, BM8 14-4801-82	IHC	1:50

Anti-Akt/PKB Antibody, PH Domain, clone SKB1	Millipore, 05-591	IF	1:500
Goat Anti-Rabbit IgG H&L (HRP)	Abcam, ab672		
Rabbit Anti-Mouse IgG H&L (HRP)	Abcam, ab6728		
Rabbit Anti-Rat IgG H&L (HRP)	Abcam, ab6734		
Rabbit Anti-Goat IgG H&L (HRP)	Abcam, ab6741		
Goat anti-Mouse IgG (H+L) Cross-Adsorbed Secondary, Alexa Fluor™ 488	Thermo Scientific, A-11001		

Supplementary Table 2. The qRT-PCR primer lists for mouse genes

Gene	Gene Bank No.	Primer	Primer Sequence
ITPR3	NM_080553.3	Forward	5'-CGTCTGGGCTTTGTGGATGTG
		Reverse	5'-TGACCTGAAGGAAGGGCAGTG
ORAI1	NM_175423.3	Forward	5'-GGAAATGGCTCGGGGACAAA
		Reverse	5'-CTAGCCCCAGCAACACAGTT
ORAI2	NM_178751.3	Forward	5'-ACACATCCCAAGAGCCACAG
		Reverse	5'-ACCCTTATTCATGCGGGGAC
PMCA2	NM_009723.6	Forward	5'_CTA GAG CCG GCA AGA CAT GG
		Reverse	5'_CTG GCA TCT ACA TGG TCG GG
SERCA1	NM_007504.2	Forward	5'-TAAAGTCCTCCACCCTCGTC
		Reverse	5'-CTAGTCAGTTGCCTTGTGCC
SERCA2	NM_001110140.3	Forward	5'-GCTTCTTCTGTAGGTCTGATGGT
		Reverse	5'-TAGGCGAAGGGACAGAAACC
STIM1	NM_009287.4	Forward	5'-CCCATCAGCATCAGCACTAC
		Reverse	5'-TGGTTCAAATATAACATCCTTGCC
TRPC1	NM_011643.3	Forward	5'-GCAAACCCGTTTTGTTCGCA
		Reverse	5'-CACTGATAGCTGCCCCTCTT
TRPM4	NM_175130.4	Forward	5'-GAAGCCCGGGATCGGATTAG
		Reverse	5'- TCAGCAACTCCTTTCGGGTC
TRPM7	NM_021450.2	Forward	5'_GCA GCT TTG TTA CCG GAT TGG
		Reverse	5'_ACG GGC TTA AAT GGA GAA GCA
CCl2	NM_011331	Forward	5'-TTAAAAACCTGGATCGGAACCAA
		Reverse	5'-GCATTAGCTTCAGATTTACGGG
F4/80	NM_153100.1	Forward	5'-GCCTATTATCTATACCCTCCAGCACATC
		Reverse	5'-TCCATCTCCCATCCTCCACATCAG
IL1B	NM_008361.4	Forward	5'-TCCATTGAGGTGGAGAGCTTTC
		Reverse	5'-CTACAGGCTCCGAGATGAACAAC
TNF-a	NM_013693	Forward	5'-AGACCCTCACACTCAGATCATCTTC
		Reverse	5'-CCTCCACTTGGTGGTTTGCT
ATF3	NM_007498.3	Forward	5'-CCTTATCATCCCGGCCAGTC
		Reverse	5'-GTCCCACCCCACCTATCAAG
ATF4	NM_009716.3	Forward	5'-CGCTCTTCACGAAATCCAGC
		Reverse	5'-TCATGTTGTGGGGCTTTGCT
ATF6	NM_001081304.1	Forward	5'-CTTGCCTTGTACGCAACTCC
		Reverse	5'-GCACTGCCACCAGAGAATTG
IRE1a	NM_023913.2	Forward	5'-GCCCTCAGCATGATGGACTT
		Reverse	5'- TGTCTTGCCTCCAAAGGATGT

XBP1	NM_013842.3	Forward	5'-ATCGCAGGGAGGGTCATTTG
		Reverse	5'-TGGGGTCAAGAGGGTCAGAA
XBP1s		Forward	5'-GAGTCCGCAGCAGGTG
		Reverse	5'-GTGTCAGAGTCCATGGGA
CEBPB	NM_009883.4	Forward	5'-GACAAGCTGAGCGACGAGTA
		Reverse	5'-GTCAGCTCCAGCACCTTGT
ChREBP	NM_021455.4	Forward	5'-ATGCGGGACATGTTTGATGA
		Reverse	5'-TCGGATGAGGATGCTGAACAC
NFkB	NM_008689	Forward	5'-TGGCAGCTCTTCTCAAAGCA
		Reverse	5'-CCCAAGAGTCGTCCAGGTCAT
NRF2	NM_010902.5	Forward	5'-GCCCTCAGCATGATGGACTT
		Reverse	5'- TGTCTTGCCTCCAAAGGATGT
Nurr1	NM_013613.2	Forward	5'-AGCTGGTTGAAGTGTCCAGT
		Reverse	5'-GCAGGTTAGGAAATAGCGACA
Nur77	NM_010444.2	Forward	5'-GGTTCCCTGGACGTTATCCG
		Reverse	5'-GGCGGAGGATGAAGAGTTCC
PPARa	NM_011144.6	Forward	5'-CGCTCCCGACCCATCTTTAG
		Reverse	5'-ACTGGGCTACATCCTCGACT
PPARd	NM_011145.3	Forward	5'-ACCAGCAGCCTAAAAGCAGT
		Reverse	5'-CCCAGAGAGAGCGTAGTGTT
PPARg	NM_011146	Forward	5'-CAGGCCGAGAAGGAGAAGCT
		Reverse	5'-GGCTCGCAGATCAGCAGACT
SREBP1c	NM_011480.3	Forward	5'-GTACCTGCGGGACAGCTTAG
		Reverse	5'-TCAGG TCATGTTGGAAACCA
G6PC	NM_008061.3	Forward	5'-GGCGCAGCAGGTGTATACTATGT
		Reverse	5'-CAAACACCGGAATCCATACGT
PEPCK	NM_011044.2	Forward	5'-CGCAAGCTGAAGAAATATGACAA
		Reverse	5'-TCGATCCTGGCCACATCTC
FABP1	NM_017399.5	Forward	5'-AAGGGAAGGACATCAAGGGG
		Reverse	5'-GGTGAACTCATTGCGGACCA
FABP4	NM_024406.4	Forward	5'-GTCACCATCCGGTCAGAGAG
		Reverse	5'-CCTGTCGTCTGCGGTGATTT
FABP5	NM_010634.3	Forward	5'-ACGGGAAGGAGAGCACGATA
		Reverse	5'-AGCCCTCATTGCACCTTCTC
CD36	NM_007643	Forward	5'-ATACAGAGTTCGTTATCTAGCCAAGGA
		Reverse	5'-CCATTGGGCTGTACAAAAGACA
Ldlr	NM_001252658.1	Forward	5'-CCCTTCTCCTTGGCCATCTAT
		Reverse	5'-GAGTCGATTGGCACTGAAAATG
Cidea	NM_007702.2	Forward	5'-GGGACAACACGCATTTCATG
		Reverse	5'-GTTGCTTGCAGACTGGGACAT
Cideb	NM_026925.3	Forward	5'-ACAGTCCGGAAAGGACTGACA

		Reverse	5'-CACTCCACGTAGCAGCAAGGT
Cidec	NM_178373.3	Forward	5'-CCATCAGAACAGCGCAAGAA
		Reverse	5'-ACTGGGCTACATCCTCGACT
Acc1	NM_133360.2	Forward	5'-CTGAAGCAGATCCGCAGCTT
		Reverse	5'-GGTGAGA TGTGCTGGGTCATG
Acc2	NM_133904.2	Forward	5'-TGGAGTCCATCTTCCTGTCC
		Reverse	5'-CGAATATGGTCGTTTCTGAAAGT
FAS	NM_007988.3	Forward	5'-GAAGAAGTGTCTGGACTGTGTCATTT
		Reverse	5'-TTAATTGTGGGATCAGGAGAGCAT
Cpt1	NM_013495.2	Forward	5'-TCGTGGTGGTGGGTGTGAT
		Reverse	5'-GGGTCCGATTGATCTTTGCA

Supplementary Table 3. The qRT-PCR primer lists for human genes

Gene	Gene Bank No.	Primer	Primer Sequence
hITPR1	NM_001099952.2	Forward	5'_CGG AGC AGG GTA TTG GAA CA
		Reverse	5'_CTG AGG GCT GAA ACT CCA GG
hITPR2	NM_002223.3	Forward	5'_CTT CAT CAG CAC CTT GGG GT
		Reverse	5'_GGC ACA CCT TGA AAA GGC AG
hITPR3	NM_002224.3	Forward	5'_GCT TCA TCA GCA CTT TGG GG
		Reverse	5'_ATG GGG CAC ACC TTG AAG AG
hORAI1	NM_032790.3	Forward	5'_AAG CTT AAA GCC TCC AGC CG
		Reverse	5'_GTG GGT AGT CGT GGT CAG C
hORAI2	NM_001271819.1	Forward	5'_ATG GGA TGG AGA GCC TGA GT
		Reverse	5'_GGT ACT GGT ACT GCG TCT CC
hPMCA2	NM_001001331.3	Forward	5'_GGT GTG AAG AAG GGG GAT GG
		Reverse	5'_CTA GGC TGG CAT TCG CAC TA
hSERCA1	NM_004320.4	Forward	5'_GTG GGA GCT GGT GAT AGA GC
		Reverse	5'_TCA CCT TCC TCA AAC CAG GC
hSERCA2	NM_001681.3	Forward	5'_TTG CTC GAG TTG AAC CCT CC
		Reverse	5'_CGG CTT TCT TCA GAG CAG GA
hSTIM1	NM_003156.3	Forward	5'_TTG TGT CTC CCT TGT CCA TGC
		Reverse	5'_GGG TCA AAT CCC TCT GAG ATC C
hTRPC1	NM_001251845.1	Forward	5'_AAG CTT TTC TTG CTG GCG TG
		Reverse	5'_CTC CCA AGC ACA TCT ACG CA
hTRPM7	NM_017672.5	Forward	5'_GAG GAG GAG GAG GTG GAG TT
		Reverse	5'_TCC CTC TTG GTC AAA GTG CT

References

1 Kim, O.-H. *et al.* Externalized phosphatidylinositides on apoptotic cells are eat-me signals recognized by CD14. *Cell Death Differ.* **29**, 1423-1432 (2022).

2 Kang, J. K. *et al.* Increased intracellular Ca2+ concentrations prevent membrane localization of PH domains through the formation of Ca2+-phosphoinositides. *Proc. Natl. Acad. Sci. U S A* **114**, 11926-11931 (2017).
